# Strategies, Barriers, and Facilitators for Healthcare Professionals to Recommend HPV Vaccination: A Systematic Review

**DOI:** 10.3390/vaccines13040402

**Published:** 2025-04-12

**Authors:** Yihan Fu, Yinqi He, Zhitao Wang, Jing Sun

**Affiliations:** School of Health Policy and Management, Chinese Academy of Medical Sciences & Peking Union Medical College, Beijing 100730, China; s2024026017@student.pumc.edu.cn (Y.F.); heyinqi02@stu.njmu.edu.cn (Y.H.); s2023026008@pumc.edu.cn (Z.W.)

**Keywords:** healthcare professionals, HPV vaccine, recommendation, vaccination, systematic review

## Abstract

**Background/Objectives**: The HPV vaccine has demonstrated efficacy in preventing approximately 90% of HPV infection-associated cancers. However, the global HPV vaccination coverage rate remains low. Healthcare professionals are trusted sources of information on vaccination. Enhancing their capacities to deliver credible information through appropriate strategies to support HPV vaccination decisions can help increase vaccination coverage. There is a gap in the comprehensive summary of the strategies, barriers, and facilitators for healthcare professionals to recommend HPV vaccination. **Methods**: This review systematically evaluated the existing literature on healthcare professionals’ strategies to recommend HPV vaccination worldwide, along with the barriers and facilitators. **Results**: A total of 96 original research articles were included in the analysis, comprising 25 qualitative studies, 67 cross-sectional quantitative studies, and four mixed-methods studies. Healthcare professionals typically recommend HPV vaccination by proactively sharing relevant information and emphasizing the benefits and necessity of vaccination. Although personalized and de-sexualized communication strategies may enable easier recommendation, these are often time-consuming and require a stronger level of knowledge and communication skills. The major barriers reported by Chinese healthcare professionals included concerns about the safety of the HPV vaccine and doubts regarding the necessity of HPV vaccination. In contrast, the main obstacles in other countries were limited working time and a lack of parental support. **Conclusions**: Enhancing external policy support and professional training for healthcare professionals is critical to improving healthcare provider willingness and the use of strategies to recommend HPV vaccination. Taking action to incorporate the HPV vaccine into the National Immunization Program is an essential pathway for expanding HPV vaccination coverage, narrowing disparities, and progressing towards the elimination of cervical cancer.

## 1. Introduction

Human papillomavirus (HPV) is one of the most common sexually transmitted infections worldwide, with high-risk types closely related to cervical cancer, other anogenital cancers (i.e., penile, vaginal, vulva, and anal cancers), and head and neck cancers (i.e., cancers of the oral cavity, oropharynx, and larynx), as well as low-risk types responsible for benign warts [1]. HPV infection-associated cancers affect approximately 625,600 women and 69,400 men worldwide annually [2], with 83% progressing to cervical cancer [3,4]. Almost all cervical cancer cases are caused by high-risk HPV [5]. Cervical cancer is a leading cause of cancer-related mortality among women and remains the primary cause of cancer death among women in 37 countries [6].

The HPV vaccine has demonstrated efficacy in preventing approximately 90% of HPV infection-associated cancers, particularly cervical cancer. In 2020, the World Health Organization (WHO) launched a global strategy to accelerate the elimination of cervical cancer as a public health problem, prioritizing girls aged 9–14 years as recipients for HPV vaccination [7]. A total of 143 member states of WHO began to provide the HPV vaccine in their national immunization schedule and services by the end of 2023. Among them, the regions of the Americas and Europe have a higher proportion of vaccination, and high-income countries are the regions with the best accessibility [8]. Although high-income countries (HICs) have seen a decline in cervical cancer incidence through rolling out HPV vaccination and cervical cancer screening [9], the global HPV vaccination coverage rate remains low, at only 27% for the first dose among girls under 15 years old in 2023, which is far below the 2030 target of 90% [10]. Low vaccination coverage is mainly associated with insufficient awareness of the significance of HPV vaccination and “vaccine hesitancy”, which refers to delay or refusal of vaccination despite the availability of services [11].

Healthcare professionals are trusted sources of information on vaccination and play a crucial role in influencing vaccination decisions. Healthcare professionals providing credible information through appropriate strategies is pivotal in shaping public vaccination decisions [12]. The WHO, in their global strategy to accelerate the elimination of cervical cancer as a public health problem, also stressed that a sufficiently sized health workforce, with staff who have an optimal mix of skills and who are competent and equitably distributed, can support the delivery of new strategies for cervical cancer prevention [13]. A study published in 2024 found significant differences in healthcare professional recommendation practices across the WHO regions, from over 90% in the European region to less than 30% in the Eastern Mediterranean region [14]. Healthcare professionals in low- and middle-income countries (LMICs) often show reluctance to recommend HPV vaccination to young women and girls [15]. A nationwide survey of Chinese hospital healthcare professionals in 2021 found that only 79% were willing to recommend HPV vaccination [16]. Enhancing the capacity of healthcare professionals to deliver credible information and help the public make informed decisions could help expand HPV vaccination coverage in LMICs.

Existing evidence demonstrates the perceptions of healthcare professionals towards HPV vaccination, their willingness to make recommendations, and the impact of healthcare professionals’ recommendations on HPV vaccination coverage. There remains a gap in the comprehensive summary of healthcare professionals’ strategies to recommend HPV vaccination, as well as the barriers and facilitators. This review systematically evaluated existing studies about the strategies, barriers, and facilitators for healthcare professionals to recommend HPV vaccination worldwide. By synthesizing and critically assessing these studies, this review aimed to improve HPV vaccination recommendation strategies among healthcare professionals in China and other LMICs, thereby expanding HPV vaccination coverage in these settings and contributing to the achievement of the 2030 target.

## 2. Materials and Methods

We conducted this systematic review following the Preferred Reporting Items for Systematic Reviews and Meta-Analyses (PRISMA) guidelines (Supplementary Material Table S1) [17].

### 2.1. Inclusion and Exclusion Criteria

This review included original empirical articles (quantitative and qualitative studies) published in peer-reviewed English or Chinese journals, which investigated the strategies used by healthcare professionals to recommend HPV vaccination and the associated factors necessary for them to make recommendations. The included articles were published between 1 January 2018 and 1 February 2025. Studies were excluded if the subjects did not include healthcare professionals, or if the findings did not address HPV vaccination recommendation strategies of healthcare professionals or the associated factors for them to make recommendations. Economic analysis, clinical trials or efficacy and safety studies of HPV vaccines, editorials or personal comments, HPV vaccination guidelines, books, conference abstracts, and other irrelevant studies that targeted other vaccines, cervical cancer screening, HIV prevention, etc., were excluded.

### 2.2. Information Sources and Search Strategy

Based on PubMed, Web of Science, The Cochrane Library, EMbase, CNKI, and Sinomed databases, a systematic search was conducted for studies on the strategies and associated factors for healthcare professionals to recommend HPV vaccination. The search strategies were tailored to the specific characteristics of different databases and included three main concepts, including healthcare professionals, HPV vaccination, and recommendations. Detailed search terms are listed in Supplementary Material Table S2. Additionally, reference lists of the included review studies were searched with the same inclusion and exclusion criteria to identify additional original research. Given the significant impact of the WHO’s 2017 updated recommendations on global HPV vaccination efforts, the publication time was limited between 2018 and 2025.

### 2.3. Study Selection and Data Extraction

YF and YH independently conducted the literature searches and screening and data extraction and cross-checked the results. Disagreements were resolved through discussions and consultations with the corresponding author. The two researchers first screened the titles and abstracts of the identified articles to exclude irrelevant articles and made the inclusion judgement by reading the full texts. The information extracted for anlysis included the article title, first author, publication year, study design, characteristics of the study subject, setting of the study, country of the sponsor institutions, healthcare professionals’ intention to recommend HPV vaccination, and recommendation strategies, as well as associated factors for healthcare professionals to recommend HPV vaccination.

### 2.4. Quality and Risk of Bias Assessment

The risks of bias of the included studies were assessed using the 2017 Joanna Briggs Institute (JBI) standardized critical appraisal checklist for cross-sectional quantitative study and qualitative study, respectively. Eight and ten evaluation indicators were adopted to assess the study design, implementation, and analysis of cross-sectional quantitative studies [18] and qualitative studies [19]. Given that the scoring system of the JBI checklist is not mature, we adopted the scoring method developed by previous studies [15,20]. One point was assigned to the criterion rated “yes” and zero points were assigned to the criterion rated as “no” or “unclear”. The maximum score is 8 points and 10 points, respectively. The quality of the cross-sectional quantitative study was rated as “high” (score ≥ 6), “medium” (score 3–5), and “low” (score ≤ 2), and the quality of the qualitative study was rated as “high” (score ≥ 8), “medium” (score 4–7), and “low” (score ≤ 3). YF and YH independently assessed the studies with the above scoring method. Divergences were resolved through discussion and consultation with the corresponding author.

### 2.5. Synthesis of Results

The findings of the included articles about the strategies used by healthcare professionals to recommend HPV vaccination and the associated factors for healthcare professionals to make recommendations were summarized, narratively synthesized, grouped, and presented in tables and figures.

## 3. Results

### 3.1. Identification of Studies

**Figure 1:**
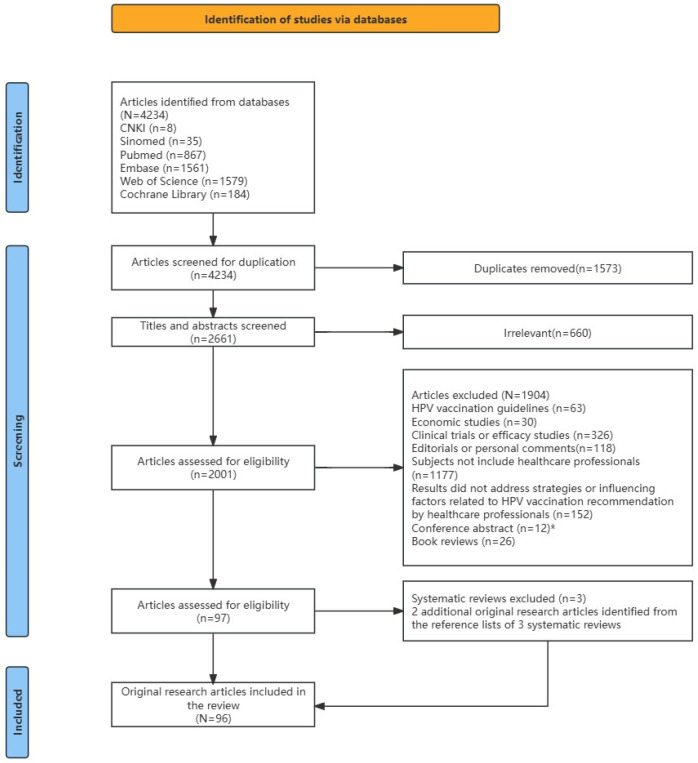
A flowchart of the article inclusion process. Note: * Among the 12 relevant conference abstracts, 3 studies were published in full articles subsequently, with different titles but the same research contents, which were included as part of the 97 articles.

We identified a total of 4234 articles, of which 2661 remained after removing duplicates. According to the exclusion criteria, a total of 2564 articles were excluded, among which two authors eliminated 660 completely irrelevant articles by screening the titles and abstracts. A total of 97 articles met the inclusion criteria, including 94 original research articles and three systematic reviews [14,21,22]. Using the same inclusion and exclusion criteria, we identified two additional original research articles from the reference lists of the three systematic reviews. Ultimately, 96 original research articles were included in the analysis (Figure 1).

### 3.2. Characteristics of Included Studies

**Figure 2:**
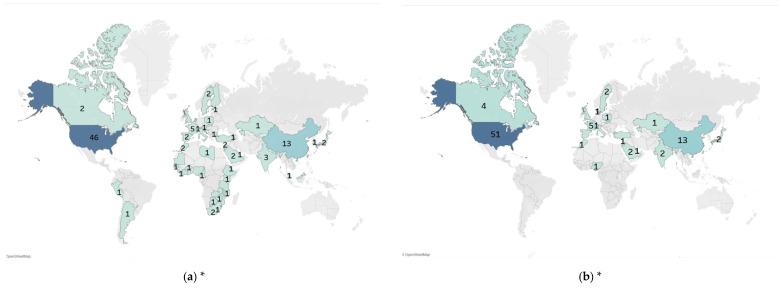
Distribution of countries of included studies (number of studies). * Numbers indicate number of studies; darker colors indicate more studies by country. (**a**) Country distribution of study subjects; (**b**) country distribution of sponsored institutions.

Among the 96 original research articles, four were mixed-methods studies. Cross-sectional quantitative studies and qualitative interviews within the same research were extracted separately, resulting in a total of 100 studies for analysis, comprising 71 cross-sectional quantitative studies and 29 qualitative interviews. Cross-sectional quantitative studies generally had larger sample sizes and varied widely from 14 to 5198 (mean = 547, median = 298). The majority of the studies were conducted in HICs, particularly in the United States (*n* = 46). Apart from China (*n* = 13), the remaining studies were conducted in the regions of Europe and Africa (Figure 2a). The sponsor institutions of 100 studies were also predominantly based in HICs, with 51 sponsored by US institutions (Figure 2b).

### 3.3. Quality Assessment

**Figure 3:**
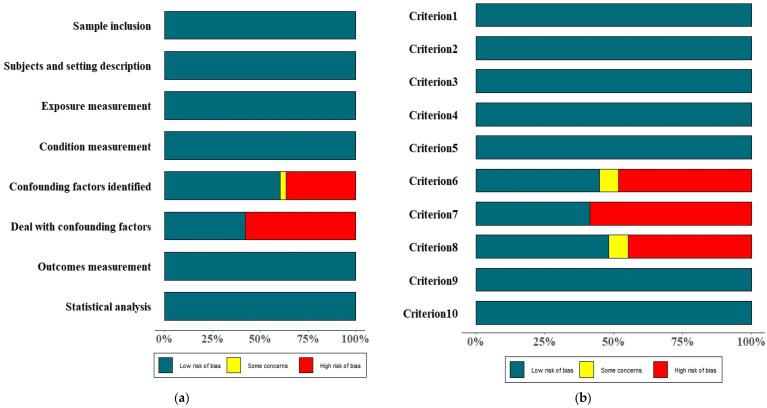
A summary of the risk of bias of the included studies. (**a**) Cross-sectional quantitative studies; (**b**) qualitative studies. Notes: Criterion 1: is there congruity between the stated philosophical perspective and the research methodology?; Criterion 2: is there congruity between the research methodology and the research question or objectives?; Criterion 3: is there congruity between the research methodology and the methods used to collect data?; Criterion 4: is there congruity between the research methodology and the representation and analysis of data?; Criterion 5: is there congruity between the research methodology and the interpretation of results?; Criterion 6: is there a statement locating the researcher culturally or theoretically?; Criterion 7: is the influence of the researcher on the research, and vice versa, addressed?; Criterion 8: are participants, and their voices, adequately represented?; Criterion 9: is the research ethical according to current criteria or, for recent studies, and is there evidence of ethical approval by an appropriate body?; Criterion 10: do the conclusions drawn in the research report flow from the analysis, or interpretation, of the data?

A total of 71 cross-sectional quantitative studies were all rated as “high” quality. The main weakness of these studies was the inadequate identification and addressing of confounding factors (Figure 3a). Among the 29 qualitative studies, 20 were rated as “high” quality, and nine were rated as “medium” quality. The common weaknesses of these studies included failure to report potential researcher bias, the social and cultural background of the researchers, and representativeness of the participants (Figure 3b).

### 3.4. Strategies Used by Healthcare Professionals to Recommend HPV Vaccination

**Figure 4:**
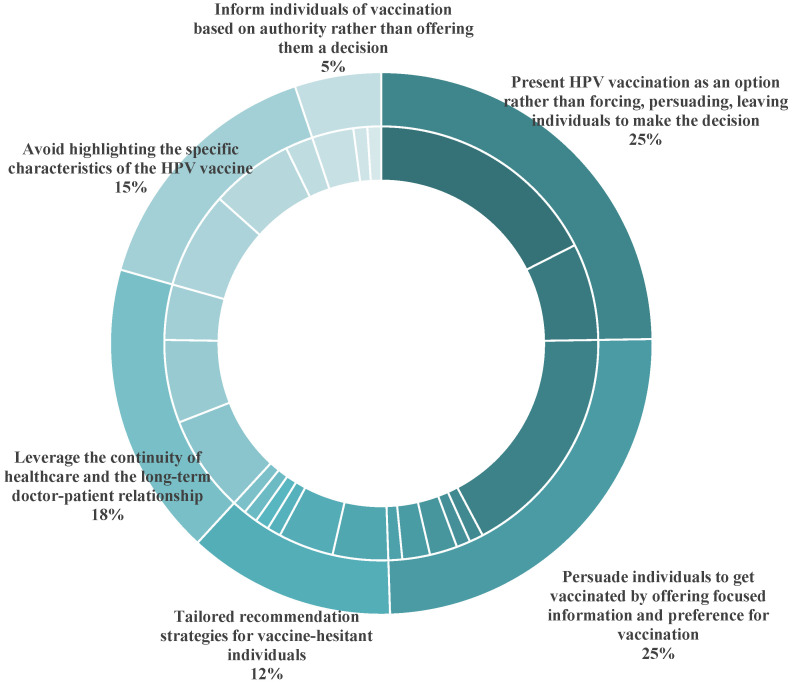
Healthcare professionals’ strategies to recommend HPV vaccination (in categories).

Among the 100 studies, 36 reported healthcare professionals’ strategies for recommending HPV vaccination, 22 of which were qualitative interviews. Three studies were conducted in LMICs, and 33 studies were conducted in HICs, primarily in the United States. No study reported the strategies used by Chinese healthcare professionals to recommend HPV vaccination. Drawing on the classification methods of previous studies [23], we divided the strategies into six categories (Figure 4) and listed 23 specific strategies in Table 1. We listed the six categories of recommendation strategies in the order of reported frequency. To facilitate the comparison of different strategies between HICs and LMICs, Table 1 also presents the number of reported studies conducted in HICs and LMICs, respectively.

The most widely employed strategy for recommending HPV vaccination by healthcare professionals globally is “presenting HPV vaccination as an option, rather than forcing, persuading, leaving individuals to make the decision” (25%). This approach includes offering patients the option of vaccination and inviting them to discuss, giving them a sense of control over the vaccine decision-making process [23,24,25,26,27,28,29,30,31,32,33,34,35,36,37], and employing motivational interviewing rather than attempting to persuade parents to accept HPV vaccination when healthcare professionals discuss concerns and fears with parents [24,29,38,39,40,41,42]. Engaging parents actively in their children’s healthcare decisions is crucial to fostering constructive dialogue with vaccine-hesitant individuals [23]. A study of Swiss healthcare professionals in 2022 highlighted that they struggled to balance providing sufficient information to guide parental decisions with respecting parental choices. Excessive promotion and strong recommendation might trigger parental skepticism or doubt about the motives of healthcare professionals, thereby interrupting the dialogue [26]. Similarly, a study of French healthcare professionals in 2024 reported that they provided expertise to families but refrained from repeated reminders, allowing families to make independent decisions [42]. An interview of Indian healthcare professionals in 2022 showed that they offered patients the option of vaccination. When parents question too much, the healthcare professionals leave it to patients and do not push further [31].

As Table 1 indicates, some healthcare professionals adopted the strategies of either emphasizing or avoiding specific aspects of HPV vaccination in the course of communications to persuade individuals get vaccinated, which were commonly employed by healthcare professionals in HICs, while only one study conducted in an LMIC reported that healthcare professionals emphasized the benefits and necessity of vaccination. Key emphasized points included the protective benefits of HPV vaccination against cervical, anal, and oral cancers, along with the economic benefits and importance of vaccination for the family [23,24,27,28,30,33,34,38,40,42,43,44,45,46,47,48,49]. Explaining the disease transmission process, outlining the preventive effects of the HPV vaccine, and emphasizing verified vaccine safety were also common strategies, which were closely related to the relevant knowledge levels of healthcare professionals [30]. Healthcare professionals in the United States also highlighted the immune-boosting effects of vaccination to stress the importance of early HPV vaccination in adolescents [43]. To avoid sensitive sexual topics, some healthcare professionals employed language techniques to normalize HPV vaccination, such as presumptive or bundled recommendations—a strategy exclusively reported by healthcare professionals in the United States [23,24,27,35,39,43,46,47,50,51,52]. Presumptive recommendations assume parental willingness and frame vaccination as a routine procedure. Healthcare professionals informed patients that the HPV vaccine should be administered right now rather than presenting it as an option. This approach streamlined the conversation and bypassed sensitive topics that might cause discomfort to patients or their parents, thereby reducing the likelihood of vaccine hesitancy [24,47]. With the bundled recommendation approach, healthcare professionals recommended HPV vaccination alongside other routine adolescent vaccines without separate distinction [43]. This technique leveraged parents’ trust in other routine vaccines to normalize HPV vaccination and avoid sensitive sexual conversations [52]. French healthcare professionals emphasized the effect of HPV vaccination in preventing cancer rather than other diseases like genital warts, intended to indirectly de-sexualize the HPV vaccine [42].

For patients who declined HPV vaccination during the visits, some healthcare professionals leveraged the continuity of healthcare and the long-term doctor–patient relationship to monitor patients’ vaccination status, and followed up through continued education as well as electronic reminders to recommend vaccination at subsequent visits [23,25,28,29,32,33,42,53,54,55,56]. Additionally, three studies reported that oncologists in the United States collaborated with primary healthcare professionals to verify patients’ vaccination status prior to cancer treatment and offered basic health education to primary healthcare teams, which extended the role of the oncologists in assisting patients in completing the course of HPV vaccination [28,55].

As Table 1 shows, tailored recommendation strategies for those who were vaccine-hesitant required additional time and advanced skills, which were only reported by a limited number of healthcare professionals in HICs. Healthcare professionals in the United States and France used personal experiences to motivate HPV vaccination [34,39,42,52] and adapted the approaches to avoid sensitive topics for minority populations, who might be culturally sensitive to sexual topics or may have language barriers. In some cases, they communicated directly with patients to deal with taboo topics, bypassing their guardians [23,34,39,47,52]. Only a very small minority of healthcare professionals informed individuals of vaccination based on authority rather than offering them a decision, such as employing authority-based strong recommendations to urge adolescents to receive HPV vaccination as early as possible, or even vaccinated the patient against HPV during the same visit alongside other routine vaccines [30,40,44,46,57]. An interview of French general practitioners, gynecologists, and pediatricians in 2018 reported that those who were firmly convinced of the reliability and necessity of vaccination usually refused to compromise, even if this resulted in the interruption of treatment [44].

### 3.5. Willingness of Healthcare Professionals to Recommend HPV Vaccination

Among the 100 studies, 96 analyzed the factors associated with healthcare professionals’ willingness to recommend HPV vaccination, including 73 international studies and 13 studies conducted in China. Among the 73 international studies, 19 were from LMICs.

#### 3.5.1. Practices of International Healthcare Professionals

**Figure 5:**
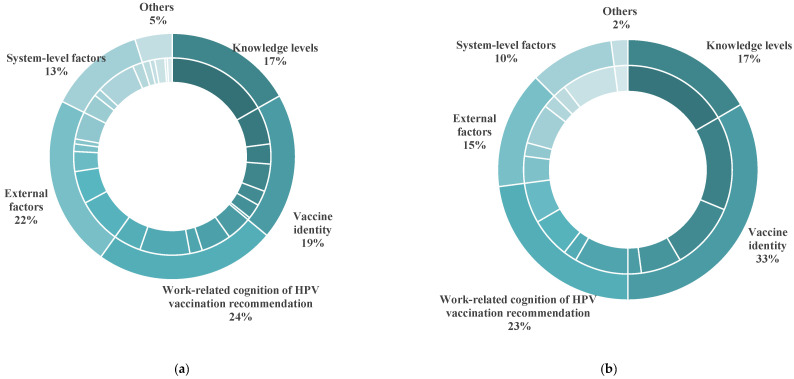
The categories of factors associated with the willingness of international and Chinese healthcare professionals to recommend HPV vaccination. (**a**) The categories of factors reported by international studies; (**b**) the categories of factors reported by studies conducted in China.

The following factors were reported by the 73 international studies, including healthcare professionals’ perceptions of recommendation for HPV vaccination (24%), external factors (22%), vaccine identity (19%), knowledge levels (17%), and system-level factors (13%). The proportion of studies that reported each type of factor among 73 studies, along with the number of studies that identified specific factors, are shown in Figure 5a and Table 2. To facilitate the comparison of results between HICs and LMICs, Table 2 presents the number of studies conducted in HICs and LMICs that reported respective factors.

Among the 73 studies that reported factors associated with international healthcare professionals’ recommendations for HPV vaccination, the most frequently mentioned factor was healthcare professionals’ perceptions of recommendation for HPV vaccination. These factors were consistently reported by studies conducted across HICs and LMICs (Table 2). These perceptions included whether healthcare professionals believed they had sufficient time to recommend HPV vaccination [23,25,27,31,39,51,53,54,55,56,58,59,60,61,62,63,64,65,66,67], their confidence in influencing patient decision-making [30,32,39,45,53,59,63,65,68,69,70,71], whether they were comfortable to discuss sex-related topics with patients or their parents [23,25,27,42,54,56,61,72,73,74], whether they believed recommending HPV vaccination was within their responsibilities [28,30,49,55,63,68,70,75,76], and whether they prioritized HPV vaccination recommendations [28,32,56,62,77]. An interview of physicians in the United States published in 2018 found that a lack of time was the most frequently cited barrier for HPV vaccination recommendation. Physicians considered it futile to argue with patients or family members who did not share the same convictions [25]. Surveys of healthcare professionals in the United States published in 2020 and 2023 also identified time availability as a significant factor associated with HPV vaccination recommendation. Many physicians noted that limited treatment time made it challenging to discuss vaccination in detail with patients or their parents, and HPV vaccination recommendation was not a priority, as their primary role was to address immediate health issues [28,65]. A survey of Turkish oncologists in 2018 found that physicians prioritized cancer treatment over HPV vaccination recommendation [57]. Many studies reported that healthcare professionals did not consider HPV vaccination recommendation as part of their role [28,30,55,57,63,68,70,75,78]. Additionally, studies also highlighted that discussing the sexually related aspects of HPV vaccination made healthcare professionals feel uneasy, which deterred them from providing recommendations [23,25,27,42,48,54,56,61,72,73,74].

External factors were also crucial, including the attitudes of patients’ parents [23,25,26,27,28,31,43,44,45,48,51,62,67,79,80,81,82] and external support for healthcare professionals [28,31,32,36,41,54,58,59,63,75,83,84,85]. “Vaccine hesitancy” is a common problem in both HICs and LMICs. Parents’ attitudes impacted not only healthcare professionals’ willingness to recommend HPV vaccination but also their manners of recommendations [28,79]. A survey of pediatricians and general practitioners from the United States in 2019 found that when parents were only concerned about vaccine safety, healthcare professionals used authoritative information to persuade patients to get vaccinated. However, if parents believed that their children were not sexually active and did not need the HPV vaccine, there was a decline in the use of authority by healthcare professionals to recommend HPV vaccination, leading to delayed vaccination [27]. Another survey of pediatricians, general practitioners, and caregivers from the United States in 2024 found that they were more likely to recommend HPV vaccination to parents who did not experience vaccination refusal than those who had such a history [51]. According to Table 2, 13 studies conducted in HICs found that a lack of information support prevented healthcare professionals from recommending HPV vaccination [28,32,36,41,54,58,59,63,75,84,85]. The support included official vaccination guidelines, advocacy materials, training, and communication tools. Three studies conducted in Switzerland, Italy, and Japan found that healthcare professionals who were informed about HPV vaccination data or official government policies were more willing to recommend HPV vaccination than those who relied on mass media [36,41,75]. This was evident in Table 2, which shows that a higher proportion of studies conducted in LMICs reported that a lack of policy support and financial incentives affected healthcare professionals’ willingness to recommend HPV vaccination compared to studies conducted in HICs [30,64,68,81]. Additionally, patients’ gender, age, and risk of sexual activity also influenced healthcare professionals’ willingness and intensity to recommend HPV vaccination [28,34,51,54,60,70,73,86,87,88,89].

Healthcare professionals’ views on the HPV vaccine directly influenced their willingness to recommend HPV vaccination. These views included perceptions of vaccine safety [25,30,41,45,61,62,63,68,74,81,85,87,90,91,92], efficacy [41,44,45,56,57,74,81,85,89,90,92], necessity [25,32,41,74,77,91,92,93], importance [45,63,84,85,94,95], concerns that HPV vaccination might lead to increased sexual activity [29,44,57,68,87,90], and reduced cervical cancer screening [42]. Safety concerns regarding the HPV vaccine were the most frequently cited vaccine identity factor for reluctance to recommend HPV vaccination. As Table 2 indicates, this issue was more severe among healthcare professionals in LMICs compared to HICs. A study conducted in 2020 in Ghana reported that a lack of awareness and evidence supporting HPV vaccine safety was the main reason for healthcare professionals not recommending HPV vaccination [62]. Similarly, an interview of Nigerian healthcare professionals in 2022 listed their beliefs in the efficacy and safety of the HPV vaccine as a key factor associated with their willingness to recommend HPV vaccination [68]. Studies conducted in both HICs and LMICs reported that healthcare professionals who believed cervical cancer screening was effective enough to identify HPV infections and doubted the efficacy of the HPV vaccine, or who were unaware of the severity of cervical cancer, were unlikely to recommend vaccination.

Healthcare professionals’ knowledge level about HPV vaccination was also a critical factor associated with their willingness to make recommendations [25,28,31,35,39,45,55,63,65,66,68,70,72,73,75,77,79,83,84,86,87,88,94,96,97,98,99,100,101,102,103], including their knowledge of HPV infection, the HPV vaccine, and communication strategies to recommend HPV vaccination. A survey of physicians in the United States published in 2018 found that 89% of them cited insufficient knowledge of the HPV vaccine as a major barrier to recommending vaccination [25]. A survey of the American Society of Gynecologic Oncology members in 2021 revealed that better knowledge of the HPV vaccine was positively associated with HPV vaccination recommendation [102]. Similarly, a survey of Japanese primary healthcare professionals in 2023 found that those with higher scores in vaccination knowledge tests were more likely to recommend HPV vaccination under voluntary conditions [101]. Similar findings were also observed in LMICs. A cross-sectional quantitative study of Lebanese physicians in 2019 showed that those with higher knowledge scores in HPV infections and the HPV vaccine encountered fewer barriers to recommending HPV vaccination [79]. An interview of Nigerian healthcare professionals in 2022 found that knowledge of the HPV vaccine affected their abilities to recommend HPV vaccination [68]. A study of Slovenian healthcare professionals in 2023 highlighted that those with inadequate training and skills had limited abilities to provide professional information and this, therefore, negatively affected their recommendations and patients’ HPV vaccination decisions [73].

International studies also identified system-level factors as the determinants for healthcare professionals to recommend HPV vaccination. This was evident in Table 2, which shows that a higher proportion of studies conducted in LMICs reported vaccine availability and affordability as the critical factors influenced healthcare professionals’ willingness to recommend HPV vaccination. In these countries, high vaccine prices, storage and distribution costs, and vaccine supply shortages were the primary system-level barriers against the recommendation of HPV vaccination. Patients generally had limited financial resources, making it crucial for healthcare professionals to consider whether patients could afford the expensive HPV vaccine [31,62,68,73,79,81,83,90,92]. The capacity of healthcare institutions to provide vaccines and vaccination services also affected healthcare professionals’ HPV vaccination recommendations [31,67,68,73,83,90]. In contrast, the main system-level factor was public funding coverage of the HPV vaccine in HICs [25,28,80,87,89]. Some surveys of healthcare professionals in the United States found that insurance coverage of HPV vaccination would promote their recommendations for HPV vaccination [25], and they were more likely to recommend HPV vaccination to adolescents aged 11–13 and 14–17 years with private insurance [87]. The reported barriers for making recommendations for HPV vaccination are summarized in Table 3.

#### 3.5.2. Practices of Chinese Healthcare Professionals

A total of 13 studies reported factors associated with the recommendations of Chinese healthcare professionals for HPV vaccination [78,104,105,106,107,108,109,110,111,112,113,114,115] and identified vaccine identity (33%) as the most critical factor that affected Chinese healthcare professionals’ tendency to recommend HPV vaccination. Their perceptions of recommendation for HPV vaccination (23%), knowledge level (17%), external factors (15%), and system-level factors (10%) were also reported (Figure 5b and Table 2). The main vaccine identity barrier for Chinese healthcare professionals was their concerns about the safety of the HPV vaccine. Surveys conducted in Beijing and three southern provinces of China found that 61% and 61.9% of healthcare professionals did not recommend HPV vaccination, respectively. Concerns about HPV vaccine safety were the key barrier [105,109]. Multiple regression analysis also found that healthcare professionals who shared concerns about the safety and risk of the HPV vaccine were unlikely to recommend vaccination [104].

In addition to vaccine identity factors, cognitive barriers among Chinese healthcare professionals regarding HPV vaccination recommendation were also noted. The issue of “uncomfortable discussion with patients or parents about sexual topics related to HPV vaccine” was more prominent in China than in other countries, probably related to the specific social and cultural background as well as the low awareness of the HPV vaccine in Chinese settings [78,112]. Other cognitive factors associated with HPV vaccination recommendation in China were similar to those in other countries, including a lack of working time. A survey of immunization services in Beijing in 2021 found that increased consultation calls, appointment registration, telephone notifications, and complaint handling following HPV vaccination might reduce healthcare professionals’ willingness to actively recommend HPV vaccination [108].

The knowledge level was also identified as an important factor that influenced Chinese healthcare professionals’ recommendations for HPV vaccination, which was consistent with the findings from studies conducted in other countries. A survey of Chinese healthcare professionals conducted in 2022 in three southern provinces showed that the knowledge level regarding the HPV vaccine was an independent factor that determined healthcare professionals’ recommendations for HPV vaccination. Higher knowledge levels were associated with a greater likelihood of recommending HPV vaccination, possibly because that higher knowledge enhanced healthcare professionals’ confidence in recommending HPV vaccination [109]. A national survey of Chinese healthcare professionals from 2023 also found that those who were aware of the HPV vaccine were more willing to recommend HPV vaccination [104].

Compared to other countries, Chinese healthcare professionals rarely reported external factors as barriers to making HPV vaccination recommendations. Two studies of healthcare professionals in Beijing and those involved in HPV vaccination in Shenzhen reported that the lack of official policy support affected Chinese healthcare professionals’ recommendations for HPV vaccination [105,113]. A survey of Chinese male nurses in 2020 mentioned the lack of scientific materials and support mechanisms for HPV vaccination for men [110]. Sensitivity around gender-related stigma might also contribute to communication discomfort among male healthcare professionals.

Like their international counterparts, Chinese healthcare professionals also considered system-level factors as barriers to HPV vaccination recommendation. Similar to most LMICs, high vaccine cost was still the primary system-level barrier for Chinese healthcare professionals to recommend HPV vaccination before 2024. The prices of locally developed HPV vaccines dramatically decreased thereafter, and the issue of insufficient HPV vaccine supply is not prominent anymore [109,111,112].

#### 3.5.3. Differences of Willingness to Recommend HPV Vaccination

Significant differences were observed globally in the willingness of various healthcare professionals to recommend HPV vaccination. Obstetricians, gynecologists, and pediatricians were more inclined and experienced in recommending HPV vaccination compared to other specialties [44,61,77,78,84,86,87,105,111,116,117]. Female and younger healthcare professionals were more likely to recommend HPV vaccination [35,49,60,71,78,79,81,91,94,105,106]. Among five studies which compared the willingness of Chinese healthcare professionals across different types of medical institutions who recommend HPV vaccination, four found that primary healthcare professionals in charge of vaccination in community health centers were more willing to recommend HPV vaccination than specialists like pediatricians, obstetricians, gynecologists, and those in charge of vaccination in hospitals [78,104,106,109]. In contrast, among seven international studies that compared the intentions of different healthcare professionals to recommend HPV vaccination, only two reported that general practitioners were more likely to recommend HPV vaccination [94,118], while the remaining five studies found that specialists like pediatricians and gynecologists were more likely to recommend HPV vaccination [44,61,77,84,87].

## 4. Discussion

### 4.1. Strategies of Healthcare Professionals for Recommending HPV Vaccination and Associated Factors Were Different in HICs and LMICs

The commonly adopted strategies used by international healthcare professionals to recommend HPV vaccination include proactively providing related information to patients and their parents, engaging parents in healthcare decision-making, emphasizing the benefits and necessity of vaccination, addressing the concerns of patients and their parents in a targeted manner, and promoting sustainable and constructive dialogue with them. These recommendation strategies are selective and easier to implement and are commonly used by healthcare professionals in all countries. However, our results showed that personalized and de-sexualized communication strategies may have higher recommendation strength and are predominantly emphasized by healthcare professionals in HICs.

The longer history of HPV vaccination in HICs gives them a better social and market basis to use personalized and de-sexualized communication strategies. HICs’ mature healthcare services and vaccination support systems provide ample scope for the promotion of HPV vaccination. Earlier access to the market, public insurance coverage, and inclusion in national immunization programs have given HPV vaccination a broad population base in HICs, and healthcare professionals have a solid knowledge of and trust in HPV vaccines, which is an intrinsic driver for the implementation of personalized and de-sexualized communication strategies.

In contrast, LMICs lack such favorable contextual conditions. This disparity manifests in two primary aspects. On the one hand, the delayed introduction of the HPV vaccine in these regions is evident. Such constrained vaccination environments significantly limit healthcare professionals’ capacity to make effective recommendations. Although the financial support of Gavi, the Vaccine Alliance, has partially alleviated the vaccination burden in some LMICs, HPV vaccination rates remain persistently low. Furthermore, LMICs face substantial challenges rooted in healthcare professionals’ limited knowledge about HPV vaccination and prevalent vaccine hesitancy, which undermine their ability and confidence in recommending the vaccine. Many healthcare professionals tend to associate HPV vaccination with the initiation of sexual activity, perceiving support for vaccinating young girls as an implicit endorsement of early sexual behavior. The WHO’s recommendation of the priority age for HPV vaccination has been wrongly regarded as the starting time of sexual activeness, instead of the age of the highest level of immune system response [119]. The lack of HPV vaccination experience and knowledge makes some of the stronger recommendations difficult to accept.

No study reported the specific communication strategies used by Chinese healthcare professionals to recommend HPV vaccination. Since the HPV vaccine has not been included in the National Immunization Program (NIP), Chinese healthcare professionals are not mandated to recommend HPV vaccination. By the end of 2024, China was not among the 143 out of 194 WHO member states that include the HPV vaccine in the NIP [120]. To keep up with the international developments, some local governments started to launch free HPV vaccination programs for junior high school girls in China. Primary healthcare professionals based in the community healthcare centers have played an important role in providing free HPV vaccination services and related health education. The dissemination of information about cervical cancer prevention and treatment, as well as the importance of HPV vaccination through brochure distribution and on-site lectures in communities and schools, are the typical strategies to recommend HPV vaccination in these pilot areas. Further communication with those who are “vaccine hesitant” at HPV vaccination sites and cervical cancer screening sites through long-term monitoring and repeated reminders is also commonly adopted by primary healthcare professionals. However, tailored communication methods based on the specific situation of the target population are rarely adopted. A digital electronic system to facilitate long-term follow-ups and reminders and the de-sexualized and uncompromising communication strategies that have been widely used by international healthcare professionals are necessary and need to be established in China.

### 4.2. Willingness of Healthcare Professionals to Recommend HPV Vaccination and Associated Factors Were Different in HICs and LMICs

The existing literature indicates that vaccine identity, external factors, perceptions of HPV vaccination recommendation, knowledge levels, and system-level factors are the primary factors that affect recommendations for HPV vaccination by healthcare professionals worldwide. According to our results, perceptions of recommendation for HPV vaccination and knowledge levels both influenced the willingness of healthcare professionals in either HICs or LMICs to recommend HPV vaccination. Healthcare professionals in HICs more often reported that their willingness to recommend HPV vaccination was hampered by a lack of information support. Healthcare professionals in LMICs more frequently reported concerns about HPV vaccine safety, a lack of policy and financial support, and the high cost and insufficient supply of the HPV vaccine as barriers to their recommendation of the HPV vaccine.

Healthcare professionals in HICs frequently cited the influence of external information on their recommendations for HPV vaccination. Some complained about a lack of sufficient informative materials to persuade patients to get vaccinated. Abundant information, particularly negative contents disseminated by social media and the Internet, hindered their willingness to recommend HPV vaccination. A study in the United States found that parents who had seen any information against HPV vaccination on social media, whether alone or mixed with information in favor, more often chose to delay or refuse vaccination compared to those who did not see any information [121]. As for healthcare professionals, Italian research found that, compared with those who did not receive any information, those who obtained information from mass media and the Internet were less likely to seek vaccination [75].

In contrast to HICs, there was a significant disparity in the propensity to recommend HPV vaccination among healthcare professionals in LMICs. This gap was primarily attributed to a low awareness of the HPV vaccine among healthcare professionals, particularly their safety concerns about the HPV vaccine. Time lag and the exclusion of the HPV vaccine from the NIPs were the commonly reported reasons behind the low vaccination coverage and awareness in most of the LMICs with low HPV vaccination coverage. More time and efforts might be needed for healthcare professionals and the general population in LMICs to understand the safety and efficacy of the HPV vaccine.

Healthcare professionals in LMICs frequently emphasized the critical impact of insufficient external support, particularly in terms of policy and financial backing, on their willingness to recommend HPV vaccination. In these nations, domestic government budgets often prove inadequate to support nationwide HPV vaccination initiatives, making multi-channel funding support particularly crucial. The support primarily comes from international organizations and vaccine manufacturers. The United Nations Children’s Fund (UNICEF) has implemented a centralized procurement mechanism that facilitates vaccine manufacturers’ adoption of tiered pricing policies. This system enables LMICs to access vaccines at preferential prices through a formal application process [122]. The Pan American Health Organization (PAHO) provides a mechanism for many Gavi-ineligible, middle-income nations in Latin America and the Caribbean to procure HPV vaccines at reduced prices [123]. In addition, two countries—Bhutan and Rwanda—introduced the HPV vaccine with donations and launched its national HPV vaccination program in 2011, delivering vaccines to schools for all girls in primary grade six [124]. With government commitment, school-based delivery, and global support, it was reported that Rwanda had reached over 98% HPV coverage and aspires to be the first country in Africa to achieve the elimination of cervical cancer [125].

Regarding system-level factors, vaccine allocation and cost remain the key issues for the expansion of HPV vaccination in LMICs, especially in sub-Saharan Africa, Central America, and Southeast Asia, where cervical cancer incidences are the highest [126]. High interest in HPV vaccination by countries across all income groups has led to a sharp increase in demand in the past several years [13]. However, a combination of factors, primarily linked to continued supply constraints, has slowed the pace of HPV vaccine introduction, particularly in low-resource settings. As a result of the adjustment of introduction plans, especially in Gavi-supported countries, and the issuing of adapted global policy recommendations along with increases in the available HPV supply, the supply–demand balance has significantly improved [124]. The uneven allocation of internal resources has also directly affected HPV vaccination coverage, which has led to the fact that vulnerable populations, including racial and ethnic minorities, those who are socioeconomically disenfranchised, and those in rural areas have lower rates of HPV vaccination [127]. In terms of vaccine costs, LMICs eligible for UNICEF/Gavi or the PAHO have access to vaccines at a centralized international purchase price. The reported price per dose of HPV vaccines shows a tiered structure by the procurement method and income group, with UNICEF and the PAHO paying the lowest prices, at USD 4.50 and USD 9.98, respectively. However, affordability remains a concern for MICs that no longer are or never were supported by Gavi or the PAHO. The self-procuring MICs’ median price for HPV2 is more than twice the UNICEF price and slightly higher than the PAHO price. Both Merck and GSK have made price commitments (under specific conditions) to countries transitioning out of Gavi support. Some countries are no longer eligible for these time-limited commitments [128].

As in other LMICs, healthcare professionals’ concerns about the safety of HPV vaccines are also an important factor that prevents healthcare professionals in China from recommending HPV vaccination. China imported the first HPV vaccine in 2016, a decade after it was launched in the United States. As of 2024, China has not included the HPV vaccine in the NIP. The delayed launch of HPV vaccines in China, coupled with their high prices since initial market entry [129], has resulted in later vaccination timings and lower vaccination rates among the Chinese population. Additionally, healthcare professionals lack specialized knowledge about HPV vaccination and practical service experience. Consequently, Chinese healthcare professionals exhibit more significant concerns regarding vaccine safety compared to their international counterparts in HICs. China should also assume the responsibility of a major power to complete the catch-up.

Chinese healthcare professionals seldom reported external factors as a barrier for recommending HPV vaccination. However, policy and financial support cannot be overlooked. Although some local governments have begun to pilot free HPV vaccination programs, financial uncertainty due to the non-institutionalization of these local pilot programs have made policy and financial support problematic. Healthcare professionals’ HPV vaccination recommendations could be more credible if they were supported by sustained and stable national policy. Official endorsement serves as the foundation for healthcare professionals to make appropriate recommendations and provide protection for them. A lack of official endorsement from the central government has made healthcare professionals more likely to consider vaccination recommendation a low priority or outside the scope of their work. Moreover, male Chinese healthcare professionals also confront the issue of inadequate information support, which may be associated with the late introduction of the HPV vaccine indicated for males in China [130].

The willingness of Chinese healthcare professionals based in hospitals to recommend HPV vaccination was found to be notably weaker than that of the primary healthcare professionals based in community health centers. This was consistent with the findings of existing studies about the disparities in knowledge levels among different groups of healthcare professionals. A survey about the knowledge level of HPV infection and pathology as well as HPV vaccination among Chinese healthcare professionals based in different types of health facilities found that healthcare professionals based in hospitals had more knowledge about HPV infection and pathology, while those based in community healthcare centers scored significantly higher for knowledge related to HPV vaccination [131]. A contrary situation was observed in other countries, where specialists in pediatrics, obstetrics, and gynecology departments were more willing to recommend HPV vaccination than general practitioners. The possible reason for such a disparity might be related to the differences of setting strategies of vaccination sites. In Chinese settings, HPV vaccination services are delivered mainly by community healthcare centers, while hospitals only play a very limited role in HPV vaccination. Few studies have reported that healthcare professionals based in tertiary hospitals recommend HPV vaccination. While in other countries, HPV vaccination services are set in hospitals, clinics, pharmacies, parking areas, and communities, where general practitioners are only one part of the multiple healthcare providers [84,132]. In fact, Chinese healthcare professionals based in hospitals are more authoritative in informing patients’ healthcare decision-making and being trusted by patients. In addition, they often have access to high-risk patients. This is the common situation in most LMICs, where their primary care systems are typically not strong. Thus, healthcare professionals based in hospitals should play a greater role in making recommendations for HPV vaccination [131], thereby helping to expand vaccination coverage in LMICs.

Similar with most LMICs, high vaccine cost was still the primary system-level barrier for Chinese healthcare professionals to recommend HPV vaccination before 2024. Since 2016 imported vaccines, such as the bivalent HPV (Cervarix, USD 262 for three doses), quadrivalent HPV (Gardasil, USD 360 for three doses), and nonavalent HPV (Gardasil 9, USD 586 for three doses) vaccines have been licensed in China with high prices and limited supply [129]. With more domestic vaccines on the market and centralized procurement negotiations in more provinces, the prices of locally developed HPV vaccines dramatically decreased thereafter, and the issue of insufficient HPV vaccine supply is not prominent anymore.

### 4.3. Policy Suggestions

LMICs mainly face four challenges, included delayed or excluded HPV vaccine integration into NIPs, low public acceptance of the vaccine, insufficient policy and financing support, and high vaccination costs. To further expand HPV vaccination coverage and promote the prevention and control of cervical cancer, LMICs need to foster a comprehensive HPV vaccination ecosystem and invest efforts in both top-level design and specific practices. On one hand, LMICs need to incorporate the HPV vaccine into the NIP with secured financial back-up. Diversified funding channels and robust centralized procurement mechanisms are critical for LMICs, particularly MICs that are no longer eligible for Gavi or the PAHO. Moreover, in resource-constrained LMICs, the implementation and scale-up of single-dose HPV vaccination regimens demonstrate significant potential as they prevent a higher number of cervical cancer cases while requiring fewer girls to be vaccinated per case prevented [133]. On the other hand, it is essential to increase the awareness and sense of responsibility towards HPV vaccination and recommendation among healthcare professionals at all levels. Enhancing healthcare professionals’ access to epidemiological data, evidence-based scientific resources, and competency-focused training programs should be prioritized. This necessitates the implementation of targeted policy frameworks with integrated financial incentivization mechanisms to ensure sustainable capacity building.

Incorporating HPV vaccines into the NIP is the most imperative strategy to accelerate HPV vaccination coverage in China. This would catalyze multisectoral collaboration to enhance public awareness and strengthen the social consensus regarding vaccine safety and efficacy, thereby boosting healthcare professionals’ confidence in recommending HPV vaccination. Furthermore, the NIP should secure predictable funding through central–local fiscal coordination and ensure sustainable resources for vaccination across regions with diverse socioeconomic developments. Additionally, capacity building should be strengthened with differential training curricula targeting healthcare professionals based in different healthcare settings and with different skills and knowledge. It is necessary to firstly increase the awareness of healthcare professionals in hospitals about HPV vaccination and enhance their communication skills for recommending HPV vaccination. For primary healthcare professionals in community healthcare centers, their knowledge about HPV infections and pathology should be strengthened through enhanced professional trainings thereby avoiding the dissemination of inaccurate information and confusion to patients [134,135].

### 4.4. Strengths and Limitations

Our systematic review offers a critical overview of healthcare professionals’ strategies to recommend HPV vaccination worldwide, along with the barriers and facilitators, providing practical insights to guide immunization program planning and execution. However, limitations must be acknowledged. First, the included studies were primarily conducted in HICs, and the evidence from LMICs is limited. This is due to the late market authorization of the HPV vaccine and its non-inclusion in the NIPs of the LMICs. As more LMICs now have the HPV vaccine registered and included in their NIPs and more studies to be conducted in LMICs, future analyses will include more balanced original research from HICs and LMICs. Additionally, the included studies had quite different study designs and sample sizes. The potential heterogeneities should be considered when interpreting and generalizing the study results. Finally, this analysis was based on published data, which could be affected by reporting and publication biases. The included studies were published between 1 January 2018 and 1 February 2025, with the full text available in English or Chinese. Considering that most of the LMICs begun their efforts and studies of HPV vaccination after the WHO updated recommendations on global HPV vaccination efforts in 2018, a broader inclusion time span would help to balance the amount of evidence from HICs and LMICs. Among the nine conference abstracts with the full text unavailable, four were conducted in the United States, two were in other HICs, and only three were in LMICs. Language restrictions excluded publications in French and Spanish; thus, potential findings from Africa and South America were excluded. The absence of these materials means that special care needs to be taken when interpreting and extrapolating the conclusions of this study.

## 5. Conclusions

In summary, healthcare professionals generally took strategies to share information related to the HPV vaccine and emphasized its benefits and necessity. Personalized and de-sexualized communication strategies, although more effective, require more time and richer knowledge and skills. Uncompromising and strong recommendations also require greater external support and firmer belief in HPV vaccination among healthcare professionals. The willingness of healthcare professionals to recommend HPV vaccination is primarily determined by their perceptions of HPV vaccination recommendation and external factors. The main factor that is associated with the willingness of Chinese healthcare professionals to recommend HPV vaccination is HPV vaccine identity. To further expand HPV vaccination coverage and promote the prevention and control of cervical cancer, LMICs need to foster a comprehensive HPV vaccination ecosystem and invest efforts in both top-level design and specific practices. On one hand, LMICs need to incorporate the HPV vaccine into the NIP, with secured financial back-up. On the other hand, it is essential to increase awareness and a sense of responsibility towards HPV vaccination and recommendation among healthcare professionals at all levels and to strengthen external support for them.

## Figures and Tables

**Table 1:** Healthcare professionals’ specific strategies for recommending HPV vaccination.

Categories of Recommendation Strategies	Specific Recommendation Strategies	Number of Reported Studies	Number of Reported Studies Conducted in HICs	Number of Reported Studies Conducted in LMICs
Present HPV vaccination as an option rather than forcing, persuading, or leaving individuals to make decisions	(a) Offer the option of vaccination and invite discussion, giving patients a sense that physicians have control over the vaccine decision-making process	17	14	3
	(b) Motivational interviewing	7	7	/
Persuade individuals to get vaccinated by offering focused information and preference for vaccination	(a) Emphasize the benefits and necessity of vaccination	17	16	1
	(b) Emphasize that vaccination is a commitment to family responsibilities	2	2	/
	(c) Emphasize the importance of vaccination	1	1	/
	(d) Emphasize vaccine innovation	1	1	/
	(e) Discuss the unpredictability of sexual behavior	2	2	/
	(f) Explain the economics of vaccination	1	1	/
Leverage the continuity of healthcare and the long-term doctor–patient relationship	(a) Continued education and recommendation at the patient’s next visit	7	7	/
	(b) Automated education and reminders via electronic systems	6	6	/
	(c) Collaborate with primary care physicians	3	3	/
Avoid highlighting the specific characteristics of the HPV vaccine	(a) Bundled recommendation	7	7	/
	(b) Assume parental consent to vaccination, include HPV vaccination as a routine step (presumptive recommendation)	6	6	/
	(c) De-sexualized discussion	2	2	/
Tailored recommendation strategies for vaccine-hesitant individuals	(a) Personal experience	4	4	/
	(b) Other tailored recommendation strategies	4	4	/
	(c) Offer official authority information to vaccine-hesitant individuals who do not trust healthcare professionals	1	1	/
	(d) Offer an alternative vaccination program	1	1	/
	(e) Use culturally sensitive language	1	1	/
	(f) Communicate directly with the patients to deal with taboo topics, bypassing the guardians	1	1	/
Inform individuals of vaccination based on authority rather than offering them a decision	(a) Strongly recommend HPV vaccination as early as possible	3	3	/
	(b) No compromise	1	1	/
	(c) Spread intimidation, remind individuals that vaccination is mandatory	1	/	1

**Table 2:** Factors associated with the willingness of international and Chinese healthcare professionals to recommend HPV vaccination.

Categories of Factors	Specific Factors	Number of International Studies Reported the Specific Factor	Number of Studies Conducted in HICs Reported the Specific Factor	Number of Studies Conducted in LMICs Reported the Specific Factor	Number of Studies Conducted in China Reported the Specific Factor
Perceptions and of recommendation for HPV vaccination	(a) Whether healthcare professionals believed they had sufficient time to recommend vaccination	20	16	4	3
	(b) Confidence in influencing patient decision-making	12	9	3	3
	(c) Whether they were comfortable discussing sex-related topics with patients or their parents	11	8	3	3
	(d) Beliefs that recommending HPV vaccination was within their responsibilities	10	8	2	4
	(e) Whether they prioritized HPV vaccination recommendations	5	3	2	/
External factors	(a) Attitudes of patients’ parents	18	15	3	2
	(b) Information support	13	13	/	1
	(c) Patient characteristics	11	6	5	1
	(d) Policy support	8	5	3	3
	(e) Evidence support	3	2	1	/
	(f) Financial incentives	2	2	/	/
Vaccine identity	(a) Vaccine safety	15	9	6	7
	(b) Vaccine efficacy	11	9	2	3
	(c) Concerns that vaccination might lead to more sexual activity	6	4	2	1
	(d) Vaccine importance	6	6	/	/
	(e) Vaccine necessity	8	6	2	5
	(f) Concerns that vaccination might lead to fewer cervical cancer screening	1	1	/	/
Knowledge level	Knowledge level	41	31	10	8
System-level factors	(a) Vaccine cost	16	9	7	4
	(b) Vaccine supply	8	2	6	1
	(c) Other system-level factors	4	4	/	/
	(d) Patient disease history	3	3	/	/
Others	(a) Peer drive	4	4	/	/
	(b) Vaccine manufacturer motivation	3	3	/	/
	(c) Vaccine benefit-risk ratio	2	2	/	/
	(d) Other vaccine priorities	1	1	/	/
	(e) Forgetting	2	2	/	/

**Table 3:** Barriers associated with the willingness of healthcare professionals to recommend HPV vaccination.

Categories of Barriers	Specific Barriers
Perceptions of recommendation for HPV vaccination	(a) Lack of time to recommend (b) Confidence in influencing patient decision-making
	(c) Vaccination recommendation not the work priority
	(d) Vaccination recommendation not part of responsibility
	(e) Not comfortable discussing sex-related topics
External factors	(a) Vaccine hesitancy of the patient or parent
	(b) Lack of information support
	(c) Lack of policy support
	(d) Lack of financial incentives
Vaccine identity	(a) Safety concerns
	(b) Concerns of increased sexual activity after vaccination
	(c) Concerns of reduced cervical cancer screening after vaccination
	(d) Doubt of the efficacy of the HPV vaccine
	(e) Doubt of the necessity and importance of HPV vaccination
Knowledge level	Insufficient knowledge of HPV infection, HPV vaccine, and communication strategies to recommend HPV vaccination
System-level factors	(a) High vaccination cost
	(b) Lack of capacity to provide affordable vaccine and vaccination services

## Data Availability

The study was based on open data; no primary data are to be shared.

## Supplementary Materials

The following supporting information can be downloaded at https://www.mdpi.com/article/10.3390/vaccines13040402/s1, Table S1: PRISMA 2020 Main Checklist; Table S2: Specific search strings; Table S3: Risk of bias appraisal of Cross-sectional Quantitative Studies; Table S4: Risk of bias appraisal of Qualitative Studies. References [23,24,25,26,27,28,29,30,31,32,33,34,35,36,37,38,39,40,41,42,43,44,45,46,47,48,49,50,51,52,53,54,55,56,57,58,59,60,61,62,63,64,65,66,67,68,69,70,71,72,73,74,75,76,77,78,79,80,81,82,83,84,85,86,87,88,89,90,91,92,93,94,95,96,97,98,99,100,101,102,103,104,105,106,107,108,109,110,111,112,113,114,115,116,117,118] are cited in the supplementary materials.

## Abbreviations

HPV	Human papilloma virus
WHO	World Health Organization
HICs	High-income countries
LMICs	Low- and middle-income countries
PRISMA	Preferred Reporting Items for Systematic reviews and Meta-Analyses
NIP	National Immunization Program
JBI	Joanna Briggs Institute
ACIP	Advisory Committee on Immunization Practices
CHIP	Children’s Health Insurance Program
ACA	The 2010 Patient Protection and Affordable Care Act
GPs	General practitioners
Gavi	Gavi, the Vaccine Alliance
MICs	Middle-income countries
LICs	Low-income countries
UNICEF	The United Nations Children’s Fund
PAHO	Pan American Health Organization
