# Supplementary material for: Strategies, Barriers, and Facilitators for Healthcare Professionals to Recommend HPV Vaccination: A Systematic Review
Source: Vaccines (Basel). 2025 Apr 12;13(4):402. doi: 10.3390/vaccines13040402 (PMC12031110; doi:10.3390/vaccines13040402)
Supplement: Supplementary file 1 [file vaccines-13-00402-s001.zip › vaccines-3526206-supplementary.pdf]

## Supplementary Material

**Table S1: PRISMA 2020 Main Checklist**

| Section and Topic | Item # | Checklist item | Location where item is reported |
|---|---|---|---|
| **TITLE** | | | |
| Title | 1 | Identify the report as a systematic review. | Page1 |
| **ABSTRACT** | | | |
| Abstract | 2 | See the PRISMA 2020 for Abstracts checklist. | Page1 |
| **INTRODUCTION** | | | |
| Rationale | 3 | Describe the rationale for the review in the context of existing knowledge. | Page1-2 |
| Objectives | 4 | Provide an explicit statement of the objective(s) or question(s) the review addresses. | Page2 |
| **METHODS** | | | |
| Eligibility criteria | 5 | Specify the inclusion and exclusion criteria for the review and how studies were grouped for the syntheses. | Page3 |
| Information sources | 6 | Specify all databases, registers, websites, organisations, reference lists and other sources searched or consulted to identify studies. Specify the date when each source was last searched or consulted. | Page3 |
| Search strategy | 7 | Present the full search strategies for all databases, registers and websites, including any filters and limits used. | Page3 & Supplementary Table S2 |
| Selection process | 8 | Specify the methods used to decide whether a study met the inclusion criteria of the review, including how many reviewers screened each record and each report retrieved, whether they worked independently, and if applicable, details of automation tools used in the process. | Page3 |
| Data collection process | 9 | Specify the methods used to collect data from reports, including how many reviewers collected data from each report, whether they worked independently, any processes for obtaining or confirming data from study investigators, and if applicable, details of automation tools used in the process. | Page3 |
| Data items | 10a | List and define all outcomes for which data were sought. Specify whether all results that were compatible with each outcome domain in each study were sought (e.g. for all measures, time points, analyses), and if not, the methods used to decide which results to collect. | Page3 & Supplementary Data_Total information |
| | 10b | List and define all other variables for which data were sought (e.g. participant and intervention characteristics, funding sources). Describe any assumptions made about any missing or unclear information. | Page3 & Supplementary Data_Total information |
| Study risk of bias assessment | 11 | Specify the methods used to assess risk of bias in the included studies, including details of the tool(s) used, how many reviewers assessed each study and whether they worked independently, and if applicable, details of automation tools used in the process. | Page3-4 |
| Effect measures | 12 | Specify for each outcome the effect measure(s) (e.g. risk ratio, mean difference) used in the synthesis or presentation of results. | NA |
| Synthesis | 13a | Describe the processes used to decide which studies were eligible for each synthesis (e.g. | Page4 |

| Section and Topic | Item # | Checklist item | Location where item is reported |
|---|---|---|---|
| methods | | tabulating the study intervention characteristics and comparing against the planned groups for each synthesis (item #5)). | |
| | 13b | Describe any methods required to prepare the data for presentation or synthesis, such as handling of missing summary statistics, or data conversions. | Page4 |
| | 13c | Describe any methods used to tabulate or visually display results of individual studies and syntheses. | Page4 |
| | 13d | Describe any methods used to synthesize results and provide a rationale for the choice(s). If meta-analysis was performed, describe the model(s), method(s) to identify the presence and extent of statistical heterogeneity, and software package(s) used. | Page4 |
| | 13e | Describe any methods used to explore possible causes of heterogeneity among study results (e.g. subgroup analysis, meta-regression). | NA |
| | 13f | Describe any sensitivity analyses conducted to assess robustness of the synthesized results. | NA |
| Reporting bias assessment | 14 | Describe any methods used to assess risk of bias due to missing results in a synthesis (arising from reporting biases). | NA |
| Certainty assessment | 15 | Describe any methods used to assess certainty (or confidence) in the body of evidence for an outcome. | NA |
| **RESULTS** | | | |
| Study selection | 16a | Describe the results of the search and selection process, from the number of records identified in the search to the number of studies included in the review, ideally using a flow diagram. | Page4 & Figure1 |
| | 16b | Cite studies that might appear to meet the inclusion criteria, but which were excluded, and explain why they were excluded. | Figure1 |
| Study characteristics | 17 | Cite each included study and present its characteristics. | Page5 & Figure2 |
| Risk of bias in studies | 18 | Present assessments of risk of bias for each included study. | Page5 & Figure3 |
| Results of individual studies | 19 | For all outcomes, present, for each study: (a) summary statistics for each group (where appropriate) and (b) an effect estimate and its precision (e.g. confidence/credible interval), ideally using structured tables or plots. | Page6-13 & Table1-2 & Figure4-5 & Supplementary Data_Total information |
| Results of syntheses | 20a | For each synthesis, briefly summarise the characteristics and risk of bias among contributing studies. | Page6-13 & Table1-2 & Figure4-5 |
| | 20b | Present results of all statistical syntheses conducted. If meta-analysis was done, present for each the summary estimate and its precision (e.g. confidence/credible interval) and measures of statistical heterogeneity. If comparing groups, describe the direction of the | Page6-13 & Table1-2 |

| Section and Topic | Item # | Checklist item | Location where item is reported |
|---|---|---|---|
| | | effect. | & Figure4-5 |
| | 20c | Present results of all investigations of possible causes of heterogeneity among study results. | NA |
| | 20d | Present results of all sensitivity analyses conducted to assess the robustness of the synthesized results. | NA |
| Reporting biases | 21 | Present assessments of risk of bias due to missing results (arising from reporting biases) for each synthesis assessed. | Page6 & Supplementary Table S3-4 |
| Certainty of evidence | 22 | Present assessments of certainty (or confidence) in the body of evidence for each outcome assessed. | Supplementary Data_Total information |
| **DISCUSSION** | | | |
| Discussion | 23a | Provide a general interpretation of the results in the context of other evidence. | Page13-17 |
| | 23b | Discuss any limitations of the evidence included in the review. | Page17 |
| | 23c | Discuss any limitations of the review processes used. | Page17 |
| | 23d | Discuss implications of the results for practice, policy, and future research. | Page13-18 |
| **OTHER INFORMATION** | | | |
| Registration and protocol | 24a | Provide registration information for the review, including register name and registration number, or state that the review was not registered. | NA |
| | 24b | Indicate where the review protocol can be accessed, or state that a protocol was not prepared. | NA |
| | 24c | Describe and explain any amendments to information provided at registration or in the protocol. | NA |
| Support | 25 | Describe sources of financial or non-financial support for the review, and the role of the funders or sponsors in the review. | Page18 |
| Competing interests | 26 | Declare any competing interests of review authors. | Page18 |
| Availability of data, code and other materials | 27 | Report which of the following are publicly available and where they can be found: template data collection forms; data extracted from included studies; data used for all analyses; analytic code; any other materials used in the review. | Page18 |

**Table S2: Specific search strings**

| | |
|---|---|
| **PubMed** | |
| 1. Healthcare professional | "Health Personnel"[MeSH Terms] OR "health personnel*"[Title/Abstract] OR "healthcare worker*"[Title/Abstract] OR "health care worker*"[Title/Abstract] OR "health care provider*"[Title/Abstract] OR "healthcare provider*"[Title/Abstract] OR "health care professional*"[Title/Abstract] OR "healthcare professional*"[Title/Abstract] OR "healthcare practitioner*"[Title/Abstract] OR "health care practitioner*"[Title/Abstract] |
| 2. HPV vaccination | "papillomavirus vaccines"[MeSH Terms] OR "papillomavirus vaccin*"[Title/Abstract] OR "hpv vaccin*"[Title/Abstract] OR "papilloma virus vaccin*"[Title/Abstract] OR cervarix[Title/Abstract] OR gardasil[Title/Abstract] OR silgard[Title/Abstract] OR cecolin[Title/Abstract] OR walrinvax[Title/Abstract] OR "inoculate papillomavirus"[Title/Abstract:~3] OR "inoculate hpv"[Title/Abstract:~3] OR "inoculate papilloma virus"[Title/Abstract:~3] OR "papillomavirus inoculation"[Title/Abstract:~3] OR "hpv inoculation"[Title/Abstract:~3] OR "papilloma virus inoculation"[Title/Abstract:~3] OR "papillomavirus immunize"[Title/Abstract:~3] OR "hpv immunize"[Title/Abstract:~3] OR "papilloma virus immunize"[Title/Abstract:~3] OR "papillomavirus immunization"[Title/Abstract:~3] OR "hpv immunization"[Title/Abstract:~3] OR "papilloma virus immunization"[Title/Abstract:~3] OR "papillomavirus immunise"[Title/Abstract:~3] OR "hpv immunise"[Title/Abstract:~3] OR "papilloma virus immunise"[Title/Abstract:~3] OR "papillomavirus immunisation"[Title/Abstract:~3] OR "hpv immunisation"[Title/Abstract:~3] OR "papilloma virus immunisation" [Title/Abstract:~3] |
| 3. Recommendation | "health communication"[MeSH Terms] OR communicat*[Title/Abstract] OR conversation*[Title/Abstract] OR dialog*[Title/Abstract] OR talk[Title/Abstract] OR "talking"[Title/Abstract] OR "information exchange"[Title/Abstract] OR recommend*[Title/Abstract] OR counsel*[Title/Abstract] OR practi?e [Title/Abstract] OR suggest*[Title/Abstract] OR advi?e[Title/Abstract] |
| 4. Combined search | #1 AND #2 AND #3 AND (2018:2025[pdat]) AND (chinese[Filter] OR english[Filter]) |
| **Web of Science** | |
| 1. Healthcare professional | TS=("healthcare personnel*" OR "health care personnel*" OR "healthcare worker*" OR "health care worker*"OR "health care provider*" OR "healthcare provider*" OR "health care professional*" OR "healthcare professional*" OR "healthcare practitioner*" OR "health care practitioner*") |
| 2. HPV vaccination | TS=((papillomavirus OR "papilloma virus" OR "HPV") NEAR/3 (vaccin* OR inoculate OR inoculation OR immuni?e OR immuni?ation)) |
| 3. Recommendation | TS=("health communication" OR recommend* OR communicat* OR conversation* OR dialog* OR talk OR talking OR "information exchange" OR counsel* OR suggest* OR advi?e) |
| 4. Combined search | (#1 AND #2 AND #3) and 2025 or 2024 or 2023 or 2022 or 2021 or 2020 or 2019 (Publication Years) and English or Chinese (Languages) |
| **Embase** | |
| 1. Healthcare professional | 'health care personnel'/exp OR 'health care practitioner*':ab,ti OR 'health care professional*':ab,ti OR 'health care provider*':ab,ti OR 'health care worker*':ab,ti OR 'health personnel*':ab,ti OR 'health profession personnel*':ab,ti OR 'health worker*':ab,ti OR 'health professional*':ab,ti OR 'health provider*':ab,ti OR 'healthcare personnel*':ab,ti OR 'healthcare practitioner*':ab,ti OR 'healthcare professional*':ab,ti OR 'healthcare provider*':ab,ti OR 'healthcare worker*':ab,ti OR 'health care personnel*':ab,ti |
| 2. HPV vaccination | ((papillomavirus OR "papilloma virus" OR HPV) NEAR/3 (vaccin* OR inoculate OR inoculation OR immuni?e OR immuni?ation)):ab,ti |
| 3. Recommendation | 'interpersonal communication'/exp OR communicat*:ti,ab OR conversation:ti,ab OR conversations:ti,ab OR dialog*:ti,ab OR talk:ti,ab OR talking:ti,ab OR "information exchange":ti,ab |

| | |
|---|---|
| | OR recommend:ti,ab OR recommending:ti,ab OR recommendation*:ti,ab OR advi?e:ti,ab OR counsel*:ti,ab OR suggest*:ti,ab |
| 4. Combined search | #1 AND #2 AND #3 AND [2018-2025]/py AND ([chinese]/lim OR [english]/lim) |
| **Cochrane Library** | |
| 1. Healthcare professional | (Health care Personnel* OR Healthcare Personnel* OR Healthcare Worker* OR Health care Worker* OR Health Care Provider* OR Healthcare Provider* OR Health Care Professional* OR Healthcare Professional* OR healthcare practitioner* OR health care practitioner*):ti,ab,kw OR MeSH descriptor: [Health Personnel] explode all trees |
| 2. HPV vaccination | ((papillomavirus OR “papilloma virus” OR HPV) near/3 (vaccin* OR inoculate OR inoculation OR immuni?e OR immuni?ation)):ti,ab,kw OR MeSH descriptor: [Papillomavirus Vaccines] explode all trees |
| 3. Recommendation | (communicat* OR conversation* OR dialog* OR talk OR talking OR "information exchange" OR recommend* OR "Health Communication" OR counsel* OR suggest* OR advi?e):ti,ab,kw OR MeSH descriptor: [Health Communication] explode all trees |
| 4. Combined search | #1 AND #2 AND #3 |
| **CNKI** | |
| 1. Healthcare professional | (SU %=医务人员) OR (TKA=保健医疗提供者) OR (TKA=医护人员) OR (TKA=专业人员) OR (TKA=医务人员) |
| 2. HPV vaccination | (SU %=乳头状瘤病毒疫苗) OR (TKA=人乳头瘤病毒疫苗) OR (TKA=HPV 疫苗) OR (TKA=人乳头状瘤病毒疫苗) OR (TKA=希瑞适) OR (TKA=佳达修) OR (TKA=馨可宁) OR (TKA=沃泽惠) |
| 3. Recommendation | (SU %=卫生信息传播) OR (SU %=卫生服务) OR (SU %=卫生保健提供) OR (TKA=交流) OR (TKA=沟通) OR (TKA=推荐) OR (TKA=建议) OR (TKA=对话) OR TKA=信息交换) |
| 4. Combined search | #1 AND #2 AND #3 AND [2018-2025] |
| **Sinomed** | |
| 1. Healthcare professional | "医务人员"[不加权:扩展] OR "保健医疗提供者"[常用字段:智能] OR "医护人员"[常用字段:智能] OR "专业人员"[常用字段:智能]OR"医务人员"[常用字段:智能] |
| 2. HPV vaccination | "乳头状瘤病毒疫苗"[不加权:扩展] OR "人乳头瘤病毒疫苗"[常用字段:智能] OR "HPV 疫苗"[常用字段:智能] OR "人乳头状瘤病毒疫苗"[常用字段:智能] OR "希瑞适"[常用字段:智能] OR "佳达修"[常用字段:智能] OR "馨可宁"[常用字段:智能] OR "沃泽惠"[常用字段:智能] |
| 3. Recommendation | "卫生信息传播"[不加权:扩展] OR "卫生服务"[不加权:扩展] OR "卫生保健提供"[不加权:扩展] OR "推荐"[常用字段:智能] OR "建议"[常用字段:智能] OR "交流"[常用字段:智能] OR "沟通"[常用字段:智能] OR "对话"[常用字段:智能] OR "信息交换"[常用字段:智能] |
| 4. Combined search | ((#3) AND (#2) AND (#1)) AND ("2025"[时间] OR "2024"[时间] OR "2023"[时间] OR "2022"[时间] OR "2021"[时间] OR "2020"[时间] OR "2019"[时间] OR "2018"[时间]) |

**Notes**：exp/ = exploded MeSH term; ti,ab,kw. = title, abstract, keywords; * = truncation of word for alternate endings; TS = title, abstract, author keywords and Keywords Plus; SU %= exploded MeSH term; TKA = title, keywords, abstract; NEAR/x = within x words, regardless of order

**Table S3: Risk of bias appraisal of Cross sectional Studies**

| Included study | Criterion | | | | | | | | Overall assessment |
|---|---|---|---|---|---|---|---|---|---|
| | 1 | 2 | 3 | 4 | 5 | 6 | 7 | 8 | |
| Tang G, et al.., et al. (2018).[1] | yes | yes | yes | yes | no | no | yes | yes | low |
| E. S. Almughais, et al.., et al. (2018).[2] | yes | yes | yes | yes | yes | yes | yes | yes | low |
| T. Çataklı, et al.., et al. (2018).[3] | yes | yes | yes | yes | yes | no | yes | yes | low |
| S. E. Dilley, et al.., et al. (2018).**[4]** | **yes** | **yes** | **yes** | **yes** | **unclear** | no | **yes** | **yes** | low |
| J. Abi Jaoude, et al.., et al. (2019).[5] | **yes** | **yes** | **yes** | **yes** | **yes** | **yes** | **yes** | **yes** | low |
| P. Lake, et al., et al. (2019)..[6] | **yes** | **yes** | **yes** | **yes** | no | no | **yes** | **yes** | low |
| Lin M, et al.., et al. (2020).[7] | yes | yes | yes | yes | no | no | yes | yes | low |
| Ren W, et al.(2020).[8] | yes | yes | yes | yes | yes | yes | yes | yes | low |
| M. Bozigar, et al.(2020).[9] | yes | yes | yes | yes | unclear | no | yes | yes | low |
| L. Jaeger, et al.(2021).[10] | yes | yes | yes | yes | yes | yes | yes | yes | low |
| Li J, et al.(2021).[11] | yes | yes | yes | yes | yes | yes | yes | yes | low |
| J. Shaw, et al.(2023).[12] | yes | yes | yes | yes | no | no | yes | yes | low |
| O. G. Chido-Amajuoyi, et al.(2024).[13] | **yes** | **yes** | **yes** | **yes** | **yes** | no | **yes** | **yes** | low |
| Liang Q, et al.(2024).[14] | yes | yes | yes | yes | yes | yes | yes | yes | low |
| Li L, et al.(2019).[15] | yes | yes | yes | yes | no | no | yes | yes | low |
| Zhang L, et al.(2023).[16] | yes | yes | yes | yes | yes | yes | yes | yes | low |
| Y. Sakanishi, et al.(2023).[17] | yes | yes | yes | yes | yes | yes | yes | yes | low |
| J. A. Jaoude, et al.(2018).[18] | yes | yes | yes | yes | yes | yes | yes | yes | low |
| H. E. Alcalá, et al.(2020).[19] | yes | yes | yes | yes | no | no | yes | yes | low |
| F. M. Balogun & O. O. Omotade (2022).[20] | yes | yes | yes | yes | no | no | yes | yes | low |
| S.Ayres, et al. (2022). [21] | yes | yes | yes | yes | no | no | yes | yes | low |
| R.Btoush, et al. (2022). [22] | yes | yes | yes | yes | no | yes | yes | yes | low |
| S. Chen, et al. (2022). [23] | yes | yes | yes | yes | yes | yes | yes | yes | low |
| J. F. Domgue, et al. (2024). [24] | yes | yes | yes | yes | no | no | yes | yes | low |
| L. Dufour, et al. (2023). [25] | yes | yes | yes | yes | yes | yes | yes | yes | low |
| M. A. Garcia, et al. (2023). [26] | yes | yes | yes | yes | no | no | yes | yes | low |
| S. Hopfer , et al. (2019).[27] | yes | yes | yes | yes | yes | yes | yes | yes | low |
| M. L. Kasting, et al. (2018).[28] | yes | yes | yes | yes | yes | yes | yes | yes | low |
| W. Y. Kong, et al. (2024).[29] | yes | yes | yes | yes | yes | yes | yes | yes | low |
| Y. Mao, et al. (2023).[30] | yes | yes | yes | yes | yes | yes | yes | yes | low |
| C. Murciano-Gamborino, et al. (2024).[31] | yes | yes | yes | yes | yes | no | yes | yes | low |
| F. Napolitano, et al. (2021).[32] | yes | yes | yes | yes | yes | yes | yes | yes | low |
| A. R. Richman, et al. (2022). [33] | yes | yes | yes | yes | yes | no | yes | yes | low |
| B. L. Rosen, et al. (2019).[34] | yes | yes | yes | yes | yes | no | yes | yes | low |
| M. Schneiter, et al. (2021).[35] | yes | yes | yes | yes | yes | no | yes | yes | low |
| H. M. Topazian, et al. (2018).[36] | yes | yes | yes | yes | yes | no | yes | yes | low |
| M. K. Schneiter, et al. (2022).[37] | yes | yes | yes | yes | no | no | yes | yes | low |
| G. Della Polla, et al. (2020).[38] | yes | yes | yes | yes | no | no | yes | yes | low |
| G. Narayana, et al. (2020).[39] | yes | yes | yes | yes | no | no | yes | yes | low |
| M.K. Brewington, et al. (2024).[40] | yes | yes | yes | yes | yes | yes | yes | yes | low |
| I. Yetik, et al. (2023).[41] | yes | yes | yes | yes | yes | no | yes | yes | low |
| W.Y. Kong, et al. (2022).[42] | yes | yes | yes | yes | no | no | yes | yes | low |
| J.K.R. Francis, et al. (2021).[43] | yes | yes | yes | yes | no | no | yes | yes | low |
| M. Shuto, et al. (2021).[44] | yes | yes | yes | yes | yes | no | yes | yes | low |

| | | | | | | | | | |
|---|---|---|---|---|---|---|---|---|---|
| L.P. Hurley, et al. (2021).[45] | yes | yes | yes | yes | yes | yes | yes | yes | low |
| C.E. Halista, et al. (2020).[46] | yes | yes | yes | yes | yes | yes | yes | yes | low |
| F. Napolitano, et al. (2018).[47] | yes | yes | yes | yes | yes | yes | yes | yes | low |
| K.Z. Apaydin, et al. (2018).[48] | yes | yes | yes | yes | no | no | yes | yes | low |
| T. Dickson, et al. (2023).[49] | yes | yes | yes | yes | no | no | yes | yes | low |
| Luke P Brennan, et al. (2022).[50] | yes | yes | yes | yes | yes | yes | yes | yes | low |
| Katie Hansen, et al. (2020).[51] | yes | yes | yes | yes | no | no | yes | yes | low |
| Ding M, et al. (2024).[52] | yes | yes | yes | yes | no | no | yes | yes | low |
| A. Yacouti, et al. (2024).[53] | yes | yes | yes | yes | yes | yes | yes | yes | low |
| F. Kassymbekova, et al. (2024).[54] | yes | yes | yes | yes | no | no | yes | yes | low |
| P. Ganeshkumar, et al. (2024).[55] | yes | yes | yes | yes | no | no | yes | yes | low |
| B. Alosaimi, et al. (2024).[56] | yes | yes | yes | yes | yes | yes | yes | yes | low |
| A. Qaqish, et al. (2023).[57] | yes | yes | yes | yes | yes | no | yes | yes | low |
| D. Song, et al. (2023).[58] | yes | yes | yes | yes | yes | yes | yes | yes | low |
| M. L. Kasting, et al. (2021).[59] | yes | yes | yes | yes | yes | no | yes | yes | low |
| A. Fernandes, et al. (2023).[60] | yes | yes | yes | yes | yes | no | yes | yes | low |
| J. Thaker, et al. (2023).[61] | yes | yes | yes | yes | no | no | yes | yes | low |
| P. Sypień, et al. (2023).[62] | yes | yes | yes | yes | yes | no | yes | yes | low |
| R. Khamisy-Farah, et al. (2019).[63] | yes | yes | yes | yes | yes | yes | yes | yes | low |
| S. S. Albayat, et al. (2024).[64] | yes | yes | yes | yes | yes | yes | yes | yes | low |
| Y. Y. Lee, et al. (2019).[65] | yes | yes | yes | yes | yes | no | yes | yes | low |
| M. Steben, et al. (2019).[66] | yes | yes | yes | yes | no | no | yes | yes | low |
| G. Kara Elitok, et al. (2020).[67] | yes | yes | yes | yes | no | no | yes | yes | low |
| S. M. Sherman, et al. (2020).[68] | yes | yes | yes | yes | yes | yes | yes | yes | low |
| J. Ma, et al. (2022).[69] | yes | yes | yes | yes | yes | yes | yes | yes | low |
| C. Habermacher, et al. (2023).[70] | yes | yes | yes | yes | no | no | yes | yes | low |
| A. Hurtaud, et al. (2024).[71] | yes | yes | yes | yes | yes | yes | yes | yes | low |

**Notes:** Criterion 1: Were the criteria for inclusion in the sample clearly defined? Criterion 2: Were the study subjects and the setting described in detail? Criterion 3: Was the exposure measured in a valid and reliable way? Criterion 4: Were objective, standard criteria used for measurement of the condition? Criterion 5: Were confounding factors identified? Criterion 6: Were strategies to deal with confounding factors stated? Criterion 7: Were the outcomes measured in a valid and reliable way? Criterion 8: Was appropriate statistical analysis used?

**Table S4: Risk of bias appraisal of Qualitative Studies**

| Included study | Criterion | | | | | | | | | | Overall assessment |
|---|---|---|---|---|---|---|---|---|---|---|---|
| | 1 | 2 | 3 | 4 | 5 | 6 | 7 | 8 | 9 | 10 | |
| S. E. Dilley, et al. (2018).[4] | yes | yes | yes | yes | yes | no | no | no | yes | yes | medium |
| A. T. Fenton, et al. (2019).[72] | yes | yes | yes | yes | yes | no | no | yes | yes | yes | low |
| D. Grace, et al. (2019).[73] | yes | yes | yes | yes | yes | no | no | yes | yes | yes | low |
| P. Lake, et al. (2019).[6] | yes | yes | yes | yes | yes | no | no | yes | yes | yes | low |
| I. Kataria, et al. (2022).[74] | yes | yes | yes | yes | yes | no | no | yes | yes | yes | low |
| E. Runngren, et al. (2022).[75] | yes | yes | yes | yes | yes | yes | yes | yes | yes | yes | low |
| O. O. Odebunmi, et al. (2024).[76] | yes | yes | yes | yes | yes | no | no | yes | yes | yes | low |
| A. Tron, et al. (2024).[77] | yes | yes | yes | yes | yes | yes | yes | yes | yes | yes | low |
| A. L. Beavis, et al. (2024).[78] | yes | yes | yes | yes | yes | no | no | yes | yes | yes | low |
| D. Filakovska Bobakova, et al. (2023).[79] | yes | yes | yes | yes | yes | yes | yes | yes | yes | yes | low |
| I. Enskar, et al. (2023).[80] | yes | yes | yes | yes | yes | yes | yes | yes | yes | yes | low |
| J. M. Garbutt, et al. (2018).[81] | yes | yes | yes | yes | yes | yes | yes | yes | yes | yes | low |
| E. M. Moya, et al. (2023).[82] | yes | yes | yes | yes | yes | no | no | yes | yes | yes | low |
| P. Agyei-Baffour, et al. (2020).[83] | yes | yes | yes | yes | yes | no | yes | no | yes | yes | medium |
| M. A. Garcia, et al. (2023).[26] | yes | yes | yes | yes | yes | no | yes | no | yes | yes | medium |
| Waters, A. R., et al. (2024).[84] | yes | yes | yes | yes | yes | no | no | no | yes | yes | medium |
| M. B. Gilkey, et al. (2020).[85] | yes | yes | yes | yes | yes | yes | yes | yes | yes | yes | low |
| M. Vu, et al. (2020).[86] | yes | yes | yes | yes | yes | yes | yes | no | yes | yes | low |
| M. Bouchez, et al. (2021).[87] | yes | yes | yes | yes | yes | yes | yes | unclear | yes | yes | low |
| C. Jackson, et al. (2022).[88] | yes | yes | yes | yes | yes | yes | yes | yes | yes | yes | low |
| M. Dionne, et al. (2023).[89] | yes | yes | yes | yes | yes | yes | no | no | yes | yes | low |
| A.J. Kacew, et al. (2022).[90] | yes | yes | yes | yes | yes | unclear | no | no | yes | yes | medium |
| B.K. Grabert, et al. (2021).[91] | yes | yes | yes | yes | yes | yes | no | no | yes | yes | low |
| J. Tsui, et al. (2021).[92] | yes | yes | yes | yes | yes | yes | no | no | yes | yes | low |
| D.L. Biancarelli, et al. (2020).[93] | yes | yes | yes | yes | yes | unclear | no | no | yes | yes | medium |
| Katie Hansen, et al. (2020).[51] | yes | yes | yes | yes | yes | no | no | no | yes | yes | medium |
| E. A Janio, et al. (2025).[94] | yes | yes | yes | yes | yes | no | no | no | yes | yes | medium |
| A. C. Llavall, et al. (2021).[95] | yes | yes | yes | yes | yes | yes | yes | unclear | yes | yes | low |
| Meagan E. Miller, et al. (2023)[96] | yes | yes | yes | yes | yes | no | no | no | yes | yes | medium |

**Notes:** Criterion 1: Is there congruity between the stated philosophical perspective and the research methodology? Criterion 2: Is there congruity between the research methodology and the research question or objectives? Criterion 3: Is there congruity between the research methodology and the methods used to collect data? Criterion 4: Is there congruity between the research methodology and the representation and analysis of data? Criterion 5: Is there congruity between the research methodology and the interpretation of results? Criterion 6: Is there a statement locating the researcher culturally or theoretically? Criterion 7: Is the influence of the researcher on the research, and vice- versa, addressed? Criterion 8: Are participants, and their voices, adequately represented? Criterion 9: 9.Is the research ethical according to current criteria or, for recent studies, and is there evidence of ethical approval by an appropriate body? Criterion 10: 10.Do the conclusions drawn in the research report flow from the analysis, or interpretation, of the data?

## References


1. Tang, G.; Zhao, B.; Hu, X.; Zhao, Q.; Li, X.; Wan, Y. Knowledge, attitude and practice about human papillomavirus vaccine among female nurses working in a tertiary hospital in Hengyang city. *Chinese Journal of Public Health* **2018**, *34*, 1413-1416.
2. Almughais, E. S.; Alfarhan, A.; Salam, M. Awareness of primary health care physicians about human papilloma virus infection and its vaccination: a cross-sectional survey from multiple clinics in Saudi Arabia. *INFECTION AND DRUG RESISTANCE* **2018**, *11*, 2257-2267.
3. Çataklı, T.; Duyan-çamurdan, A.; Aksakal-Baran, F. N.; Güven, A. E.; Beyazova, U. Attitudes of physicians concerning vaccines not included in the national immunization schedule. *Turkish Journal of Pediatrics* **2018**, *60*, 290-297.
4. Dilley, S. E.; Peral, S.; Straughn, J. M.; Scarinci, I. C. The challenge of HPV vaccination uptake and opportunities for solutions: Lessons learned from Alabama. *Preventive Medicine* **2018**, *113*, 124-131.
5. Abi Jaoude, J.; Saad, H.; Farha, L.; Dagher, H.; Khair, D.; Kaafarani, M. A.; Jamaluddine, Z.; Cherfan, P. Barriers, Attitudes and Clinical Approach of Lebanese Physicians Towards HPV Vaccination; A Cross- Sectional Study. *Asian Pacific journal of cancer prevention : APJCP* **2019**, *20*, 3181-3187.
6. Lake, P.; Kasting, M. L.; Malo, T.; Giuliano, A. R.; Vadaparampil, S. T. An environmental scan to examine stakeholder perspectives on human papillomavirus vaccination: A mixed methods study. *Vaccine* **2019**, *37*, 187-194.
7. Lin, M.; Zhang, T. Current status of knowledge, attitude and practice on human papillomavirus and its vaccine among medical professionals in Beijing. *Practical Preventive Medicine* **2020**, *27*, 734-736.
8. Ren, W.; Gao, J.; Hu, Q. Influencing factors of the willingness of male nurses to HPV vaccination. *Chinese Journal of Viral Diseases* **2020**, *10*, 204-208.
9. Bozigar, M.; Faith, T. D.; White, A. A.; Drayton, K. l. D.; Fabick, A.; Cartmell, K. B. A Cross-Sectional Survey to Evaluate Potential for Partnering With School Nurses to Promote Human Papillomavirus Vaccination. *PREVENTING CHRONIC DISEASE* **2020**, *17*, E111.
10. Jaeger, L.; Senn, O.; Rosemann, T.; Plate, A. Awareness, Attitudes and Clinical Practices Regarding Human Papillomavirus Vaccination among General Practitioners and Pediatricians in Switzerland. *VACCINES* **2021**, *9*, 332.
11. Li, J.; Li, X.; Zhang, Z.; Zhao, D.; Pan, J.; Lu, L.; Wu, J. HPV vaccine-related recommendation and vaccination behavior among medical staff and attendees of immunization clinics in Beijing city. *Chinese Journal of Public Health* **2021**, *37*, 1737-1741.
12. Shaw, J.; Hanley, S.; Sitnik, E.; Berry, W.; Blatt, S.; Seserman, M.; Formica, M. K. Attitudes towards HPV Vaccination Policy Strategies to Improve Adolescent Vaccination Coverage among Pediatric Providers in New York State. *Vaccines (Basel)* **2023**, *11*, 1359.
13. Chido-Amajuoyi, O. G.; Osaghae, I.; Onyeaka, H. K.; Shete, S. Barriers to the assessment and recommendation of HPV vaccination among healthcare providers in Texas. *Vaccine: X* **2024**, *18*, 100471.
14. Liang, Q.; Qin, C.; Zhou, Y.; Shen, W.; Yang, S.; Zhao, Y.; Jiang, S.; Zhang, H. Healthcare workers' willingness to receive and recommend human papillomavirus vaccination: a cross-sectional study in Guilin city in 2023. *Chinese Journal of Vaccines and Immunization* **2024**, *30*, 149-154.
15. Li, L.; Lv, Z.; Zhang, J.; Wang, W. Survey on awareness of HPV related knowledge and vaccine among medical workers, Hekou district, Dongying city, 2019. *Preventive Medicine Tribune* **2019**, *25*, 506-509,513.
16. Zhang, L.; Deng, H.; Mao, Y.; Wang, F.; Li, J.; Zheng, P. Analysis on the influencing factors of medical workers' willingness to recommend HPV vaccine. *Fudan University Journal of Medical Sciences* **2023**, *50*, 40-47.
17. Sakanishi, Y.; Takeuchi, J.; Suganaga, R.; Nakayama, K.; Nishioka, Y.; Chiba, H.; Kishi, T.; MacHino, A.; Mastumura, M.; Okada, T.; Suzuki, T. Association between administration or recommendation of the human papillomavirus vaccine and primary care physicians' knowledge about vaccination during proactive recommendation suspension: a nationwide cross-sectional study in Japan. *BMJ Open* **2023**, *13*, e074305.

18. Jaoude, J. A.; Khair, D.; Dagher, H.; Saad, H.; Cherfan, P.; Kaafarani, M. A.; Jamaluddine, Z.; Ghattas, H. Factors associated with Human Papilloma Virus (HPV) vaccine recommendation by physicians in Lebanon, a cross-sectional study. *VACCINE* **2018**, *36*, 7562-7567.
19. Alcalá, H. E.; Maxwell, G. L.; Lindsay, B.; Keim-Malpass, J.; Mitchell, E. M.; Balkrishnan, R. Examining HPV Vaccination Practices and Differences Among Providers in Virginia. *Journal of cancer education : the official journal of the American Association for Cancer Education* **2020**, *35*, 159-164.
20. Balogun, F. M.; Omotade, O. O. Facilitators and barriers of healthcare workers' recommendation of HPV vaccine for adolescents in Nigeria: views through the lens of theoretical domains framework. *BMC health services research* **2022**, *22*, 824.
21. Ayres, S.; Gee, A.; Kim, S.; Hashibe, M.; Praag, A.; Kaiser, D.; Chang, C. P.; Brandt, H. M.; Kepka, D. Human Papillomavirus Vaccination Knowledge, Barriers, and Recommendations Among Healthcare Provider Groups in the Western United States. *Journal of cancer education : the official journal of the American Association for Cancer Education* **2022**, *37*, 1816-1823.
22. Btoush, R.; Kohler, R. K.; Carmody, D. P.; Hudson, S. V.; Tsui, J. Factors that Influence Healthcare Provider Recommendation of HPV Vaccination. *American journal of health promotion : AJHP* **2022**, *36*, 1152-1161.
23. Chen, S.; Mei, C.; Huang, W.; Liu, P.; Wang, H.; Lin, W.; Yuan, S.; Wang, Y. Human papillomavirus vaccination related knowledge, and recommendations among healthcare providers in Southern China: a cross-sectional survey. *BMC Women's Health* **2022**, *22*, 169.
24. Domgue, J. F.; Dille, I.; Kapambwe, S.; Yu, R.; Gnangnon, F.; Chinula, L.; Murenzi, G.; Mbatani, N.; Pande, M.; Sidibe, F.; et al. HPV vaccination in Africa in the COVID-19 era: a cross-sectional survey of healthcare providers' knowledge, training, and recommendation practices. *FRONTIERS IN PUBLIC HEALTH* **2024**, *12*, 1343064.
25. Dufour, L.; Carrouel, F.; Dussart, C. Human Papillomaviruses in Adolescents: Knowledge, Attitudes, and Practices of Pharmacists Regarding Virus and Vaccination in France. *Viruses* **2023**, *15*, 778.
26. Garcia, M. A.; Schlecht, N. F.; Rokitka, D. A.; Attwood, K. M.; Rodriguez, E. M. Examining the Barriers and Opportunities for Human Papillomavirus Vaccine Delivery in Cancer Care Settings: A Mixed-Methods Study. *CANCER PREVENTION RESEARCH* **2023**, *16*, 581-590.
27. Hopfer, S.; Wright, M. E.; Pellman, H.; Wasserman, R.; Fiks, A. G. HPV vaccine recommendation profiles among a national network of pediatric practitioners: understanding contributors to parental vaccine hesitancy and acceptance. *Human Vaccines and Immunotherapeutics* **2019**, *15*, 1776-1783.
28. Kasting, M. L.; Christy, S. M.; Sutton, S. K.; Lake, P.; Malo, T. L.; Roetzheim, R. G.; Schechtman, T.; Zimet, G. D.; Walkosz, B. J.; Salmon, D.; et al. Florida physicians' reported use of AFIX-based strategies for human papillomavirus vaccination. *PREVENTIVE MEDICINE* **2018**, *116*, 143-149.
29. Kong, W. Y.; Queen, T. L.; O'Shea, N. G.; Heisler-MacKinnon, J.; Liu, A.; Ozawa, S.; Brewer, N. T.; Gilkey, M. B. Impact of visit characteristics on intention to recommend HPV vaccine: An experiment with US health care professionals. *PREVENTIVE MEDICINE* **2024**, *179*, 107841.
30. Mao, Y.; Zhao, Y.; Zhang, L.; Li, J.; Abdullah, A. S.; Zheng, P.; Wang, F. Frequency of health care provider recommendations for HPV vaccination: a survey in three large cities in China. *Frontiers in public health* **2023**, *11*, 1203610.
31. Murciano-Gamborino, C.; Diez-Domingo, J.; Fons-Martinez, J.; Consortium, P. E. Healthcare Professionals' Perspectives on HPV Recommendations: Themes of Interest to Different Population Groups and Strategies for Approaching Them. *VACCINES* **2024**, *12*, 748.
32. Napolitano, F.; Pelullo, C. P.; Polla, G. D.; Angelillo, I. F. Hpv vaccination attitudes and behaviors among general practitioners in italy. *Vaccines* **2021**, *9*, 1-10.
33. Richman, A. R.; Torres, E.; Wu, Q.; Eldridge, D.; Lawson, L. HPV vaccine recommendation practices of current and future physicians in North Carolina: an exploratory study. *Health education research* **2022**, *37*, 213-226.

34. Rosen, B. L.; Rhodes, D.; Visker, J.; Cox, C.; Banez, J. C.; Lasser, B. Factors Associated with School Nurses' and Personnel's Professional Practice to Encourage Parents to Vaccinate Against Human Papillomavirus. *The Journal of school health* **2019**, *89*, 569-577.
35. Schneiter, M.; Rositch, A.; Levinson, K.; Stone, R.; Fader, A.; Ferriss, J.; Wethington, S.; Beavis, A. The gynecologic oncologist as the HPV champion: missed opportunities for cancer prevention. *Gynecologic Oncology* **2021**, *162*, S292-S293.
36. Topazian, H. M.; Kundu, D.; Peebles, K.; Ramos, S.; Morgan, K.; Kim, C. J.; Richter, K. L.; Brewer, N. T.; Peris, M.; Smith, J. S. HPV Vaccination Recommendation Practices among Adolescent Health Care Providers in 5 Countries. *Journal of Pediatric and Adolescent Gynecology* **2018**, *31*, 575-582.e572.
37. Schneiter, M. K.; Levinson, K.; Rositch, A. F.; Stone, R. L.; Nickles Fader, A.; Stuart Ferriss, J.; Wethington, S. L.; Beavis, A. L. Gynecologic oncology HPV vaccination practice patterns: Investigating practice barriers, knowledge gaps and opportunities for maximizing cervical cancer prevention. *Gynecologic Oncology Reports* **2022**, *40*, 100952.
38. Della Polla, G.; Napolitano, F.; Pelullo, C. P.; De Simone, C.; Lambiase, C.; Angelillo, I. F. Investigating knowledge, attitudes, and practices regarding vaccinations of community pharmacists in Italy. *Hum Vaccin Immunother* **2020**, *16*, 2422-2428.
39. Narayana, G.; Suchitra, J.; Kavya Suma, G.; Deepthi, G. N.; Divya Jyothi, C.; Pradeep Kumar, B. Physician’s Knowledge, Attitude, and Practice towards Human Papilloma Virus (HPV) Vaccine Recommendation in Anantapur District, Andhra Pradesh, India. *Archives of Pharmacy Practice* **2020**, *11*, 137-144.
40. Brewington, M. K.; Queen, T. L.; Heisler-MacKinnon, J.; Calo, W. A.; Weaver, S.; Barry, C.; Kong, W. Y.; Kennedy, K. L.; Shea, C. M.; Gilkey, M. B. Who are vaccine champions and what implementation strategies do they use to improve adolescent HPV vaccination? Findings from a national survey of primary care professionals. *Implement Sci Commun* **2024**, *5*, 28.
41. Yetik, I.; Tanoglu, F. B.; Pasin, O.; Cetin, C.; Ozcan, P. Knowledge levels and community guidance of doctors working in family health centers on HPV screening and HPV vaccination. *Journal of Obstetrics and Gynaecology Research* **2023**, *49*, 2519-2527.
42. Kong, W. Y.; Huang, Q.; Thompson, P.; Grabert, B. K.; Brewer, N. T.; Gilkey, M. B. Recommending Human Papillomavirus Vaccination at Age 9: A National Survey of Primary Care Professionals. *Academic Pediatrics* **2022**, *22*, 573-580.
43. Francis, J. K. R.; Rodriguez, S. A.; Dorsey, O.; Blackwell, J.-M.; Balasubramanian, B. A.; Kale, N.; Day, P.; Preston, S. M.; Thompson, E. L.; Pruitt, S. L.; Tiro, J. A. Provider perspectives on communication and dismissal policies with HPV vaccine hesitant parents. *PREVENTIVE MEDICINE REPORTS* **2021**, *24*, 101562.
44. Shuto, M.; Kim, Y.; Okuyama, K.; Ouchi, K.; Ueichi, H.; Nnadi, C.; Larson, H. J.; Perez, G.; Sasaki, S. Understanding confidence in the human papillomavirus vaccine in Japan: a web-based survey of mothers, female adolescents, and healthcare professionals. *Human Vaccines and Immunotherapeutics* **2021**, *17*, 3102-3112.
45. Hurley, L. P.; O'Leary, S. T.; Markowitz, L. E.; Crane, L. A.; Cataldi, J. R.; Brtnikova, M.; Beaty, B. L.; Gorman, C.; Meites, E.; Lindley, M. C.; Kempe, A. US primary care physicians' viewpoints on HPV vaccination for adults 27 to 45 years. *Journal of the American Board of Family Medicine* **2021**, *34*, 162-170.
46. Halista, C. E.; Kline, R. J.; Bepko, J. Understanding Barriers to HPV Vaccination: Perspectives From Air Force Family Medicine Physicians and Active Duty Air Force Males. *Military medicine* **2020**, *185*, e878-e886.
47. Napolitano, F.; Navaro, M.; Vezzosi, L.; Santagati, G.; Angelillo, I. F. Primary care pediatricians' attitudes and practice towards HPV vaccination: A nationwide survey in Italy. *PLoS One* **2018**, *13*, e0194920.
48. Apaydin, K. Z.; Fontenot, H. B.; Shtasel, D. L.; Mayer, K. H.; Keuroghlian, A. S. Primary Care Provider Practices and Perceptions Regarding HPV Vaccination and Anal Cancer Screening at a Boston Community Health Center. *Journal of community health* **2018**, *43*, 792-801.
49. Dickson, T.; Hirko, K. A.; Ford, S. Provider Confidence and Perceived Barriers when Recommending the Human Papillomavirus Vaccine to Parents. *Journal of cancer education : the official journal of the American Association for Cancer Education* **2023**, *38*, 1193-1199.

50. Brennan LP, R. N., Head KJ, Zimet GD, Kasting ML. . Obstetrician/gynecologists' HPV vaccination recommendations among women and girls 26 and younger. . *Prev Med Rep.* **2022**, *27*.
51. Hansen K, W. M., Avashia S, Duc J, Spielberg F. . What Impacts HPV Vaccination Recommendations? An Exploration of Medical Residents' Knowledge, Training, Barriers, and Practices. . *Fam Med* **2020**, *52*, 745-751.
52. Ding, M.; Xu, Z.; Lei, Q.; Wang, M.; Zhang, B.; Yang, J.; Cai, Z.; Qiao, Y.; Wang, Y. HPV vaccination knowledge and recommendation behavior among healthcare workers and teachers in Ordos city: a across-sectional analysis. *Chinese Journal of Public Health* **2024**, *40*, 625-631.
53. Yacouti, A.; Baddou, R.; Bourissi, H.; Ez-Zaouy, S.; Amayou, H.; Elmalki, K.; Got, A. E.; Benider, A.; Assoumou, S. Z.; Mouallif, M. Human PapillomaVirus Vaccine Uptake: Attitudes and Practices Among Moroccan Physicians. *Journal of cancer education : the official journal of the American Association for Cancer Education* **2024**, *39*, 588-596.
54. Kassymbekova, F.; Rommel, A.; Kaidarova, D.; Auyezova, A.; Nukusheva, S.; Dunenova, G.; Bolatbekova, R.; Zhetpisbayeva, I.; Abdushukurova, G.; Glushkova, N. Developing HPV Vaccination Communication Strategies: Assessing Knowledge, Attitudes, and Barriers Among Healthcare Professionals in Kazakhstan. *Vaccines* **2024**, *12*, 1225.
55. Ganeshkumar, P.; Tank, J.; Choudhury, S. S.; Acharya, V.; Gaur, Y.; Srivastava, R.; Janakiraman, R.; Ganeshkumar, A. Roadmap to Success: Illustrating Insights from a KAP Study on Cervical Cancer Prevention and HPV Vaccination. *South Asian Journal of Cancer* **2024**, n. pag.
56. Alosaimi, B.; Fallatah, D. I.; Abd ElHafeez, S.; Saleeb, M.; Alshanbari, H. M.; Awadalla, M.; Ahram, M.; Khalil, M. A. Predictors of Human Papillomavirus (HPV) Vaccine Acceptability Among Physicians, Their Knowledge on Cervical Cancer, and Factors Influencing Their Decision to Recommend It. *J Multidiscip Healthc* **2024**, *17*, 5177-5188.
57. Qaqish, A.; Abdo, N.; Abbas, M. M.; Saadeh, N.; Alkhateeb, M.; Msameh, R.; Tarawneh, S.; Al-Masri, M. Awareness and knowledge of physicians and residents on the non-sexual routes of human papilloma virus (HPV) infection and their perspectives on anti-HPV vaccination in Jordan. *PLOS ONE* **2023**, *18*, e0291643.
58. Song, D.; Liu, P.; Wu, D.; Zhao, F.; Wang, Y.; Zhang, Y. Knowledge and Attitudes towards Human Papillomavirus Vaccination (HPV) among Healthcare Providers Involved in the Governmental Free HPV Vaccination Program in Shenzhen, Southern China. *Vaccines (Basel)* **2023**, *11*, 997.
59. Kasting, M. L.; Head, K. J.; DeMaria, A. L.; Neuman, M. K.; Russell, A. L.; Robertson, S. E.; Rouse, C. E.; Zimet, G. D. A National Survey of Obstetrician/Gynecologists' Knowledge, Attitudes, and Beliefs Regarding Adult Human Papillomavirus Vaccination. *J Womens Health (Larchmt)* **2021**, *30*, 1476-1484.
60. Fernandes, A.; Wang, D.; Domachowske, J. B. B.; Suryadevara, M. HPV vaccine knowledge, attitudes, and practices among New York State medical providers, dentists, and pharmacists. *HUMAN VACCINES & IMMUNOTHERAPEUTICS* **2023**, *19*, 2219185.
61. Thaker, J.; Albers, A. N.; Newcomer, S. R. Nurses' perceptions, experiences, and practices regarding human papillomavirus vaccination: results from a cross-sectional survey in Montana. *BMC Nurs* **2023**, *22*, 211.
62. Sypień, P.; Marek, W.; Zielonka, T. M. Awareness and Attitude of Polish Gynecologists and General Practitioners towards Human Papillomavirus Vaccinations. *Healthcare (Basel)* **2023**, *11*, 1076.
63. Khamisy-Farah, R.; Adawi, M.; Jeries-Ghantous, H.; Bornstein, J.; Farah, R.; Bragazzi, N. L.; Odeh, M. Knowledge of human papillomavirus (HPV), attitudes and practices towards anti-HPV vaccination among Israeli pediatricians, gynecologists, and internal medicine doctors: Development and validation of an ad hoc questionnaire. *Vaccines* **2019**, *7*, 157.
64. Albayat, S. S.; Mundodan, J. M.; Elmardi, K.; Hasnain, S.; Khogali, H.; Baaboura, R.; Al-Romaihi, H. E.; AlKubaisi, N. J.; Bougmiza, M. I. Knowledge, attitude, and practices regarding human papilloma virus vaccination among physicians in Qatar. *Women's Health* **2024**, *20*.
65. Lee, Y. Y.; Wang, Z. Facilitators and barriers for healthcare providers to recommend HPV vaccination to attendees of public sexually transmitted diseases clinics in Hong Kong, China. *PLOS ONE* **2019**, *14*.
66. Steben, M.; Durand, N.; Guichon, J. R.; Greenwald, Z. R.; McFaul, S.; Blake, J. A National Survey of Canadian Physicians on HPV: Knowledge, Barriers, and Preventive Practices. *J Obstet Gynaecol Can* **2019**, *41*, 599-607.e593.

67. Kara Elitok, G.; Bulbul, L.; Altuntas, S. B.; Altuntas, B.; Günindi, G.; Haltaş, M.; Yuvarlan, A.; Toprak, D.; Bulbul, A. Recommending immunizations to adolescents in Turkey: a study of the knowledge, attitude, and practices of physicians. *Human Vaccines and Immunotherapeutics* **2020**, *16*, 1132-1138.
68. Sherman, S. M.; Cohen, C. R.; Denison, H. J.; Bromhead, C.; Patel, H. A survey of knowledge, attitudes and awareness of the human papillomavirus among healthcare professionals across the UK. *European journal of public health* **2020**, *30*, 10-16.
69. Ma, J.; Zhang, X.; Wang, W.; Zhang, R.; Du, M.; Shan, L.; Li, Y.; Wang, X.; Liu, Y.; Zhang, W.; et al. Knowledge of HPV, its vaccines, and attitudes toward HPV vaccines among obstetrician-gynecologists, pediatricians and immunization services providers in Western China. *Human Vaccines and Immunotherapeutics* **2022**, *18*, 1-7.
70. Habermacher, C.; Lalloué, B.; Lamouille, V.; Thilly, N.; Agrinier, N. Family physicians' practices and attitudes towards HPV vaccination since extension of HPV vaccination to males. *Infect Dis Now* **2023**, *53*, 104669.
71. Hurtaud, A.; Tara, A. A.; Bouazzi, L.; Pacquelet, Y.; Boiteux-Chabrier, M.; Pham, B. N.; Pierre Cavard, H.; Barbe, C. Practices of French General Practitioners Regarding Vaccination of Boys Against Human Papillomavirus (HPV), One Year After the Application of Its Official Recommendation. *J Cancer Educ* **2024**, *39*, 271-278.
72. Fenton, A. T. Abandoning Medical Authority: When Medical Professionals Confront Stigmatized Adolescent Sex and the Human Papillomavirus (HPV) Vaccine. *Journal of health and social behavior* **2019**, *60*, 240-256.
73. Grace, D.; Gaspar, M.; Rosenes, R.; Grewal, R.; Burchell, A. N.; Grennan, T.; Salit, I. E. Economic barriers, evidentiary gaps, and ethical conundrums: A qualitative study of physicians' challenges recommending HPV vaccination to older gay, bisexual, and other men who have sex with men. *International Journal for Equity in Health* **2019**, *18*, 159.
74. Kataria, I.; Siddiqui, M.; Treiman, K.; Foley, S.; Anand, M.; Biswas, S.; Shastri, D.; Bhatla, N.; Radhakrishnan, D.; Mamidi, P.; Sankaranarayanan, R. Awareness, perceptions, and choices of physicians pertaining to human papillomavirus (HPV) vaccination in India: A formative research study. *Vaccine: X* **2022**, *12*, 100228.
75. Runngren, E.; Eriksson, M.; Blomberg, K. Balancing Between Being Proactive and Neutral: School Nurses' Experiences of Offering Human Papilloma Virus Vaccination to Girls. *The Journal of school nursing : the official publication of the National Association of School Nurses* **2022**, *38*, 270-278.
76. Odebunmi, O. O.; Spees, L. P.; Biddell, C. B.; Yemeke, T.; Yanguela, J.; Higgins, C.; Gilkey, M.; Ozawa, S.; Wheeler, S. B. Benefits, challenges, and strategies related to using presumptive recommendations for HPV vaccination: A qualitative study with rural and non-rural-serving primary care professionals. *Human Vaccines and Immunotherapeutics* **2024**, *20*, 2347018.
77. Tron, A.; Schlegel, V.; Pinot, J.; Bruel, S.; Ecollan, M.; Bel, J. L.; Rossignol, L.; Gauchet, A.; Gagneux-Brunon, A.; Mueller, J.; et al. Barriers and facilitators to the HPV vaccine: a multicenter qualitative study of French general practitioners. *ARCHIVES OF PUBLIC HEALTH* **2024**, *82*, 2.
78. Beavis, A. L.; Krishnamoorthi, M. S.; Adler, S.; Fleszar, L. G.; Moran, M. B.; Rositch, A. F. Contemporary provider perspectives on how to address HPV vaccine hesitancy in the US: A qualitative study. *Vaccine X* **2024**, *20*, 100533.
79. Filakovska Bobakova, D.; Plavnicka, J.; Urbancikova, I.; Edelstein, M.; Jansen, D.; Dankulincova Veselska, Z. Barriers to HPV vaccination in marginalized Roma communities in Slovakia. *Front Public Health* **2023**, *11*, 1239963.
80. Enskar, I.; Enskar, K.; Neveus, T.; Engstrom, A. H.; Grandahl, M. Barriers in the School-Based Pan-Gender HPV Vaccination Program in Sweden: Healthcare Providers' Perspective. *VACCINES* **2023**, *11*, 310.
81. Garbutt, J. M.; Dodd, S.; Walling, E.; Lee, A. A.; Kulka, K.; Lobb, R. Barriers and facilitators to HPV vaccination in primary care practices: a mixed methods study using the Consolidated Framework for Implementation Research. *BMC family practice* **2018**, *19*, 53.
82. Moya, E. M.; Garcia, A.; Joyce Ponder, A.; Frietze, G. Addressing knowledge gaps: the key role of community health workers and healthcare providers in human papillomavirus prevention and vaccine uptake in a border community. *FRONTIERS IN PUBLIC HEALTH* **2023**, *11*, 1243539.
83. Agyei-Baffour, P.; Asare, M.; Lanning, B.; Koranteng, A.; Millan, C.; Commeh, M. E.; Montealegre, J. R.; Mamudu, H. M. Human papillomavirus vaccination practices and perceptions among Ghanaian Healthcare Providers: A qualitative study based on multi-theory model. *PLoS One* **2020**, *15*, e0240657.

84. Waters, A. R.; Weir, C.; Kramer, H. S.; van Thiel Berghuijs, K. M.; Wu, Y.; Kepka, D.; Kirchhoff, A. C. Implementation barriers and considerations for recommending and administering the human papillomavirus (HPV) vaccination in oncology settings. *Journal of cancer survivorship : research and practice* **2024**, *18*, 1481-1491.
85. Gilkey, M. B.; Grabert, B. K.; Malo, T. L.; Hall, M. E.; Brewer, N. T. Physicians' rhetorical strategies for motivating HPV vaccination. *Social Science and Medicine* **2020**, *266*, 113441.
86. Vu, M.; King, A. R.; Jang, H. M.; Bednarczyk, R. A. Practice-, provider- and patient-level facilitators of and barriers to HPV vaccine promotion and uptake in Georgia: a qualitative study of healthcare providers' perspectives. *Health education research* **2020**, *35*, 512-523.
87. Bouchez, M.; Ward, J. K.; Bocquier, A.; Benamouzig, D.; Peretti-Watel, P.; Seror, V.; Verger, P. Physicians' decision processes about the HPV vaccine: a qualitative study. *Vaccine* **2021**, *39*, 521-528.
88. Jackson, C.; Nielsen, S. M.; Simonyan, B.; Kirakosyan, M.; Hovhannisyan, M.; Sahakyan, G.; Habersaat, K. B. Medical specialists' attitudes and practices towards childhood vaccination: a qualitative study in Armenia. *BMC Pediatrics* **2022**, *22*, 620.
89. Dionne, M.; Sauvageau, C.; Kiely, M.; Rathwell, M.; Bandara, T.; Neudorf, C.; Dubé, È. "The problem is not lack of information": A qualitative study of parents and school nurses' perceptions of barriers and potential solutions for HPV vaccination in schools. *Vaccine* **2023**, *41*, 6654-6660.
90. Kacew, A. J.; Jacobson, S.; Sheade, J.; Patel, A. A.; Hlubocky, F. J.; Lee, N. K.; Henderson, T. O.; Schneider, J. A.; Strohbehn, G. W. Provider-Level Barriers to Human Papillomavirus Vaccination in Survivors of Childhood and Young Adult Cancers. *Journal of Adolescent and Young Adult Oncology* **2022**, *11*, 284-289.
91. Grabert, B. K.; Heisler-MacKinnon, J.; Liu, A.; Margolis, M. A.; Cox, E. D.; Gilkey, M. B. Prioritizing and implementing HPV vaccination quality improvement programs in healthcare systems: the perspective of quality improvement leaders. *HUMAN VACCINES & IMMUNOTHERAPEUTICS* **2021**, *17*, 3577-3586.
92. Tsui, J.; Vincent, A.; Anuforo, B.; Btoush, R.; Crabtree, B. F. Understanding primary care physician perspectives on recommending HPV vaccination and addressing vaccine hesitancy. *Human Vaccines and Immunotherapeutics* **2021**, *17*, 1961-1967.
93. Biancarelli, D. L.; Drainoni, M. L.; Perkins, R. B. Provider Experience Recommending HPV Vaccination Before Age 11 Years. *Journal of Pediatrics* **2020**, *217*, 92-97.
94. Janio, E. A.; Walker, C.; Steere, E.; Seaman, A. T.; Askelson, N.; Pagedar, N. A. A Qualitative Study of Attitudes Toward HPV Vaccine Recommendation in Otolaryngology Clinics. *Laryngoscope Investigative Otolaryngology* **2025**, *10*, e70085.
95. Llavall, A. C.; de Wildt, G.; Meza, G.; Tattsbridge, J.; Jones, L. Nurses' and teachers' perceived barriers and facilitators to the uptake of the Human Papilloma Virus (HPV) vaccination program in Iquitos, Peru: A qualitative study. *PLoS ONE* **2021**, *16*, e0255218.
96. Miller, M. E.; Rahim, M. Q.; Coven, S. L.; Jacob, S. A.; Zimet, G. D.; Meagher, C. G.; Ott, M. A. Pediatric hematology and oncology physician and nurse practitioner views of the HPV vaccine and barriers to administration. *Human Vaccines and Immunotherapeutics* **2023**, *19*.
